# Light activation of 3D-printed structures: from millimeter to sub-micrometer scale

**DOI:** 10.1515/nanoph-2021-0652

**Published:** 2022-01-11

**Authors:** Hoon Yeub Jeong, Soo-Chan An, Young Chul Jun

**Affiliations:** Department of Materials Science and Engineering, Ulsan National Institute of Science and Technology (UNIST), Ulsan 44919, Republic of Korea

**Keywords:** 3D printing, 4D printing, light activation, nanomaterial composites, photothermal activation, shape transformation

## Abstract

Three-dimensional (3D) printing enables the fabrication of complex, highly customizable structures, which are difficult to fabricate using conventional fabrication methods. Recently, the concept of four-dimensional (4D) printing has emerged, which adds active and responsive functions to 3D-printed structures. Deployable or adaptive structures with desired structural and functional changes can be fabricated using 4D printing; thus, 4D printing can be applied to actuators, soft robots, sensors, medical devices, and active and reconfigurable photonic devices. The shape of 3D-printed structures can be transformed in response to external stimuli, such as heat, light, electric and magnetic fields, and humidity. Light has unique advantages as a stimulus for active devices because it can remotely and selectively induce structural changes. There have been studies on the light activation of nanomaterial composites, but they were limited to rather simple planar structures. Recently, the light activation of 3D-printed complex structures has attracted increasing attention. However, there has been no comprehensive review of this emerging topic yet. In this paper, we present a comprehensive review of the light activation of 3D-printed structures. First, we introduce representative smart materials and general shape-changing mechanisms in 4D printing. Then, we focus on the design and recent demonstration of remote light activation, particularly detailing photothermal activations based on nanomaterial composites. We explain the light activation of 3D-printed structures from the millimeter to sub-micrometer scale.

## 1 Introduction

Three-dimensional (3D) printing is a bottom-up fabrication method used for building arbitrary 3D objects layer-by-layer with a high level of customization [1], [2], [3]. It can allow fabrication of complex structures, such as hollow or embedded multi-material structures, without post assembly. Unprecedented complexities can be realized in shapes and materials. While conventional lithography techniques, such as photolithography, are mainly limited to planar patterning, 3D printing enables the fabrication and integration of complex shapes along the out-of-plane direction. Additionally, it permits printing on nonplanar surfaces or curvilinear substrates. The application of 3D printing is rapidly increasing in various fields, including automotive, robotics, microfluidics, biomedical engineering, and photonics [4], [5], [6], [7], [8], [9], [10], [11], [12], [13], [14], [15], [16], [17].

Various materials, such as polymers, ceramics, and metal powders, can be 3D-printed at high resolutions. However, material requirements and resolutions differ across printing methods. Table 1 summarizes the main features of the major 3D-printing methods. Direct ink writing (DIW) directly extrudes inks using the shear-thinning effect. During printing, inks flow smoothly from the extruder. The printed structure maintains its shape owing to the high viscosity of the inks in the absence of shear stress. Post-crosslinking may be necessary for some polymer materials. Many materials can be printed using DIW. However, it has resolution and rigidity issues. Fused deposition modeling (FDM) is also based on material extrusion. Thermoplastic materials are melted and extruded through a nozzle and then solidified after extrusion. It is widely used in both low-cost and professional 3D printers, but it often results in low surface quality and low resolution. Selective laser sintering (SLS) involves the use of metal or ceramic powders. The powders are sintered by a high-power laser and piled up layer-by-layer to form metal or ceramic 3D structures.

**Table 1:** Types of 3D printing and its characteristics.

Printing method	Materials	Surface finish	Supports	Resolution range	Advantages	Disadvantages
FDM	Thermoplastic polymers	Standard	Yes	50–200 μm	Simple cheap High speed Versatile	Limitation in complexity Mechanically weak Limited materials Layer-by-Layer finish
PolyJet (material jetting)	Photocurable polymer	Excellent	Yes	5–200 μm	Multi-material High quality High speed	High cost
SLA/DLP	Photocurable resin	Excellent	Yes	10 μm	Recyclable raw material High quality	Single material Limited material Resin absorbs moisture
DLW	Photoresist photocurable polymer	Excellent	No	< 1 μm	Recyclable raw material	High cost Limited material Time consuming
SLS	Metal powder, ceramic powder, polymer powder	Standard	No	80–250 μm	Recyclable raw material High quality	High cost Post processing
DIW	Polymers, ceramics	Standard	No	50–200 μm	Diverse material	Low resolution
					Versatility	Fragile
					Flexible	Post curing

Stereolithography (SLA) and digital light processing (DLP) use photocurable liquid resins. SLA solidifies liquid resins through laser illumination, while DLP solidifies each layer all at once using a projector. They produce smooth surfaces, but printing materials are limited. PolyJet printing uses liquid photopolymers that are dropped from a nozzle and cured with ultraviolet (UV) light. Multi-material 3D printing can be readily realized using this method, but usually at a high cost. Direct laser writing (DLW) uses an ultrafast laser to induce nonlinear multi-photon absorption in a small laser spot and solidify materials. Multi-photon absorption has an advantage over single-photon absorption in terms of resolution. DLW can achieve resolutions down to the sub-micrometer scale, which makes it widely usable for 3D microstructure patterning [18, 19].

Recently, a new concept has emerged in 3D printing known as four-dimensional (4D) printing [20]. Components that are 3D printed are usually static structures with fixed shapes and functions. However, 4D printing adds active and responsive functions to 3D-printed structures. This can be realized, for example, by printing with smart materials such as shape memory polymers (SMPs), liquid crystal elastomers (LCEs), and hydrogels. 4D-printed structures can respond to environmental stimuli, where active responses are programmed into materials via structural and compositional design. Adaptive or reconfigurable structures with desired structural and/or functional changes can be realized through 4D printing. Potential applications include actuators, soft robots, sensors, medical devices, and active photonic devices and components. The rapid development of multi-material 3D printing has accelerated 4D printing research. Multi-material printing can play a key role in realizing various active structures. Based on multi-material 3D printing, various studies have been conducted on multifunctional structures and sequentially deforming or actuating structures.

**Figure 1:**
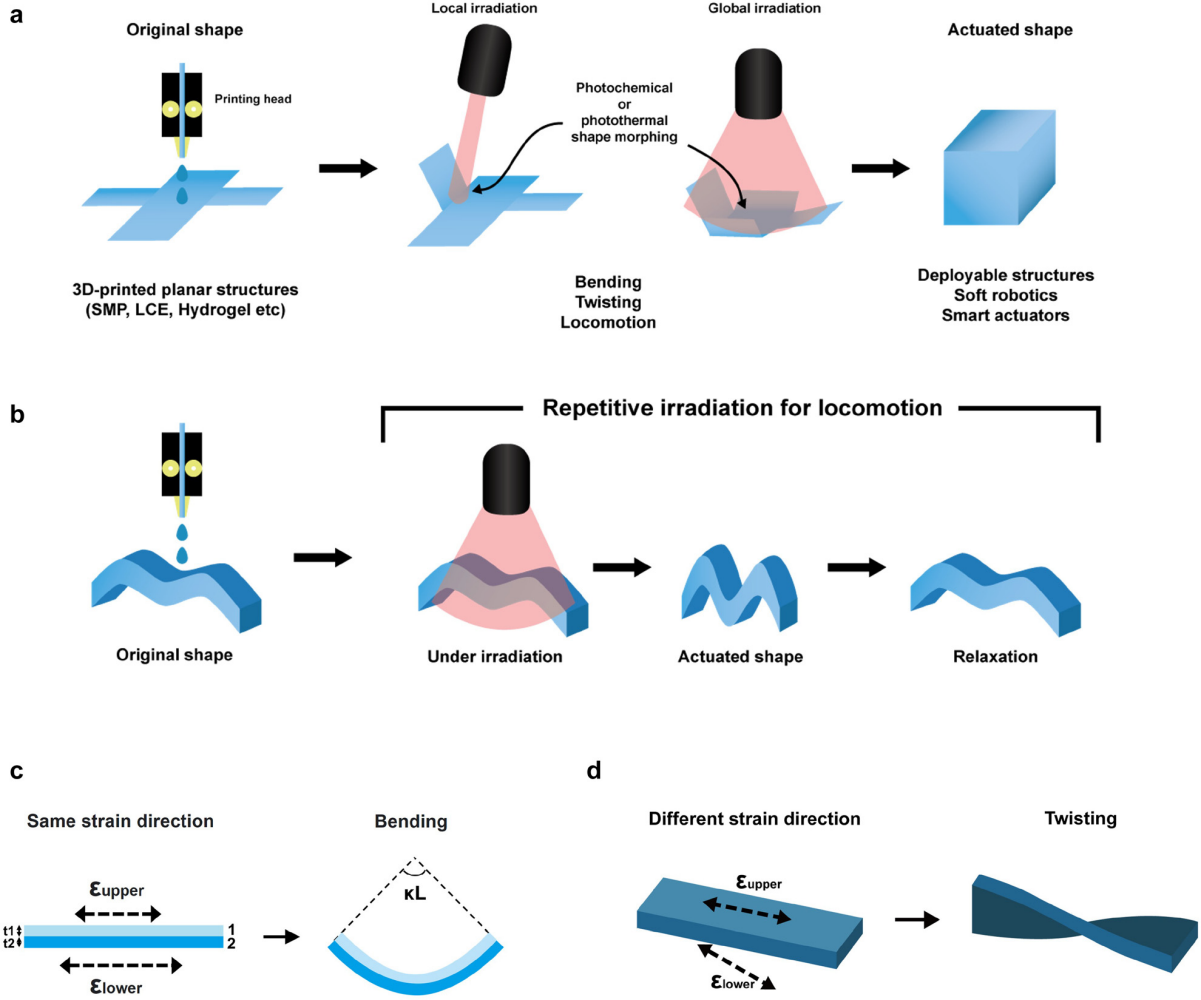
General concept for the light activation of 3D-printed structures. (a) 3D-printed planar structures can be transformed into 3D structures under local or global light illumination. Deployable or adaptive structures can be realized with desired structural and functional changes. (b) 3D-printed nonplanar structures can be transformed into other 3D structures under light illumination. Light-induced locomotion can be realized by repetitive light irradiation. (c) Bending of a bilayer structure. The thicknesses of the upper and lower layers are t_1_ and t_2_, respectively, and the length is L. (d) Twisting of a bilayer structure.

The shape or properties of 3D-printed structures can be transformed in response to external stimuli such as heat, light, humidity, pH, and electric or magnetic fields [21], [22], [23], [24], [25], [26], [27], [28], [29], [30]. Compared to other external stimuli, light has unique advantages (Figure 1a and b). It can induce structural or functional changes remotely and selectively. Laser light can also be focused to a small spot to enable local activation with a high resolution in space and time. Various properties of light (e.g., intensity, polarization, wavelength) can be rapidly and precisely adjusted to control the response of printed structures.

Here, we introduce designs and principles for light activation (Figure 1). There are two main mechanisms for light activation: photochemical and photothermal activations. There have been studies on the light activation of nanomaterial composites, but they were limited to rather simple planar structures. More recently, the light activation of 3D-printed complex structures has attracted increasing attention. However, there has been no comprehensive review of this emerging research topic yet. In this paper, we present a comprehensive review of the 3D printing of light-activated structures. We first introduce representative smart materials (Section 2) and shape transformation procedures (Section 3) in general 4D printing. Then, we discuss the design and recent demonstration of remote light activation (Section 4). Specifically, we discuss photochemical and photothermal activations, particularly detailing photothermal activations based on nanomaterial composites. We describe how such light-activation mechanisms were used in the recent demonstrations of 3D-printed, active structures from the millimeter scale to the sub-micrometer scale (Sections 4.3–4.5). Finally, we conclude in Section 5.

While our review aims to discuss recent progresses in 3D-printed structures, we also included discussions on general photochemical (Section 4.1.1) and photothermal (Section 4.2) activations based on traditional fabrication methods (such as hot pressing and molding) [31, 32]. These sections provide general backgrounds for the following sections (Sections 4.1.2 and 4.3–4.5) on the light activation of 3D-printed structures.

## 2 Representative smart materials for 4D printing

**Figure 2:**
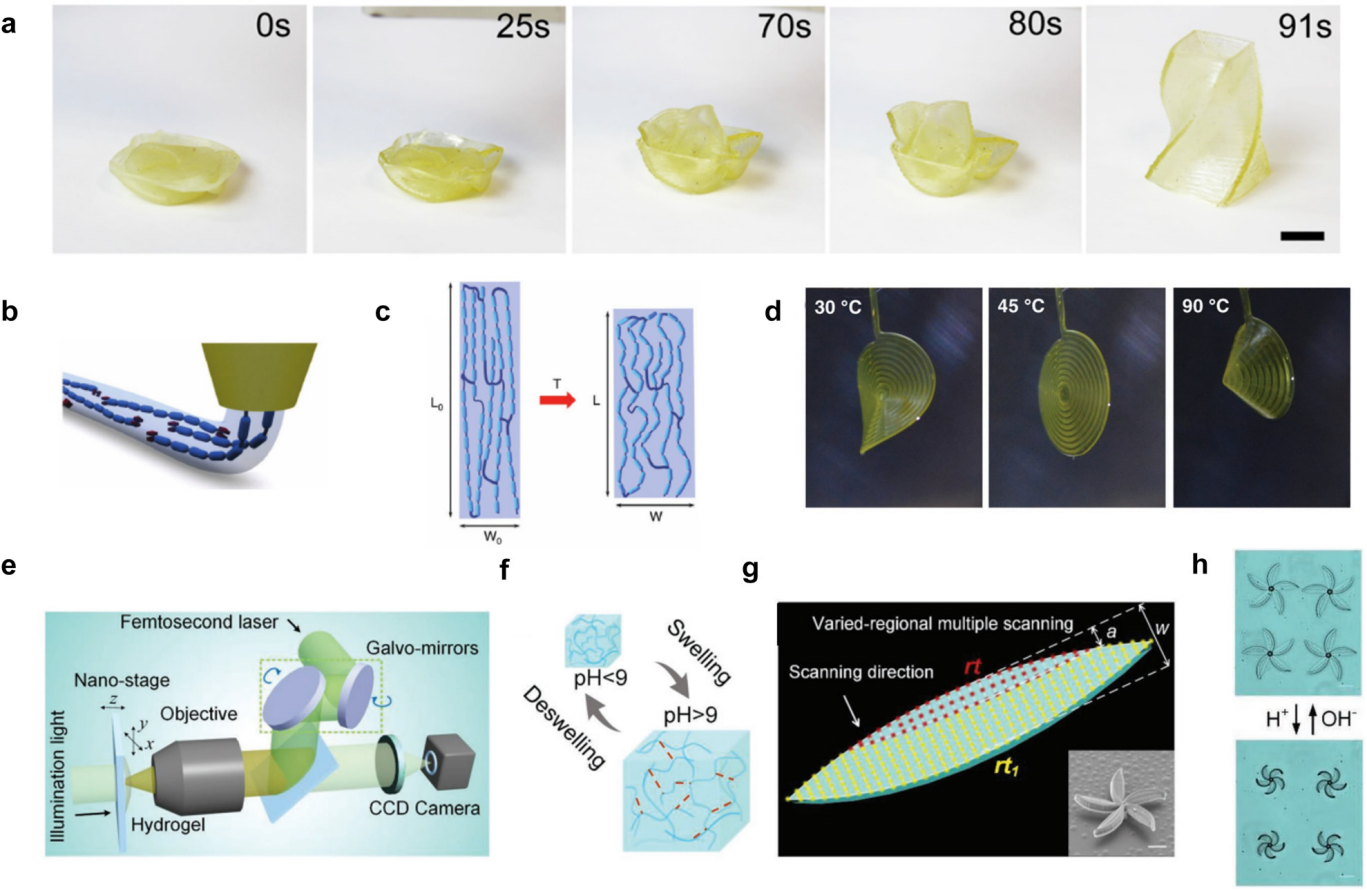
Representative smart materials for 4D printing. (a) Shape recovery of a 3D-printed SMP vase structure. Adapted from Ref. [41]. Copyright (2018) ACS Publications. (b) Alignment of LC mesogens during printing. (c) Shape morphing of a printed LCE strip upon temperature variation. (d) Shape morphing of a printed spiral structure. Adapted from Ref. [45]. Copyright (2017) John Wiley & Sons. (e) A schematic of the DLW setup for microstructure printing. (f) The swelling and deswelling of printed hydrogels depend on pH level. (g) Schematic for varied-regional multiple scanning for the chiral actuation of petals in a botanical structure. The inset is an SEM image of the printed botanical structure. Scale bar: 20 μm. (h) Chiral actuation of printed botanical structures upon pH level variation. Adapted from Ref. [52]. Copyright (2020) John Wiley & Sons.

Printing with smart materials can be used to achieve 4D printing. SMPs are one of the most widely used smart materials for 4D printing [33], [34], [35], [36], [37], [38], [39], [40]. They are composed of a solid part and deformable molecular chains. SMPs, which are rigid at room temperature, can have a transition from rigid to rubbery states above a specific temperature called the glass transition temperature *T*_g_. Therefore, a temporary shape can be formed above *T*_g_ and fixed by cooling it back to room temperature. This procedure to create a fixed, temporary shape is often called *thermomechanical* programming. The original shape is recovered by shape memory properties when heated above *T*_g_ again because the locked molecular chains become mobile above *T*_g_. If an SMP sample is stretched during thermomechanical programming, the shape fixity ratio (*R*_f_) can be defined as *R*_f_ = $\frac{\epsilon_{\text{f}}-\Delta \epsilon }{\epsilon_{\text{f}}}$ , where $\epsilon_{\text{f}}$ is the fixed strain value of the sample before releasing programming force and Δϵ is the strain change after releasing the programming force. The shape recovery ratio (*R*_r_) can be defined as *R*_r_ = $1-\frac{\epsilon_{\text{t}}}{\epsilon_{\text{r}}}$ , where $\epsilon_{\text{r}}$ is the programmed strain value and $\epsilon_{\text{t}}$ is the time-dependent strain value. For example, Kuang et al. demonstrated the DIW of thermally-activated structures using self-healing SMPs (Figure 2a) [41]. A 3D-printed vase structure was compressed above *T*_g_ and fixed in a temporary, flat shape. Then, the recovery of the original vase structure was demonstrated at an increased temperature.

Liquid crystals (LCs) have two different phases: aligned and isotropic states. Different alignments of LCs result in different refractive indices. Likewise, LCEs are polymers that have aligned and isotropic states. Different alignments of mesogens (i.e., compounds exhibiting LC properties) result in different lengths. Typically, LCEs are printed in an aligned state along the printing path. Above a certain critical temperature, the alignment of the LCEs is broken. Consequently, the LCEs return to the isotropic state and are shortened in length. Because the transition between aligned and isotropic states is reversible, many 4D printing studies have been conducted using LCEs [42], [43], [44]. For example, López-Valdeolivas et al. demonstrated 4D-printed actuators using LCEs (Figure 2b–d) [45]. LC mesogens were aligned during printing (Figure 2b), and LCEs were crosslinked by UV light exposure after printing. Figure 2c and d shows the shape morphing of the printed structure. The aligned state at room temperature was broken at high temperature, inducing the shape transformation.

Hydrogels are smart materials that are frequently used in 4D printing research and respond to various external stimuli [46], [47], [48], [49], [50]. For example, shape deformation can be achieved by the swelling of hydrogel structures under external stimuli. Bakarich et al. demonstrated shape morphing structures with temperature-sensitive hydrogels [51]. The swelling ratio of alginate/poly(N-isopropylacrylamide) hydrogels can be varied with temperature. At room temperature, the swelling ratio was about 9%. However, it decreased to under 2% at 60 °C. Utilizing variable swelling factors, a thermally-activated hydrogel valve was demonstrated. Hu et al. demonstrated the DLW of 4D-printed microscale botanical structures using pH-sensitive hydrogels (Figure 2e–h) [52]. A pH-sensitive hydrogel precursor was crosslinked using a femtosecond laser (Figure 2e). The swelling of hydrogels depends on pH level (Figure 2f). In addition, the swelling ratio of hydrogels can be controlled by the degree of crosslinking. Using these features, a chiral actuation of printed botanical structures was demonstrated upon the pH level variation (Figure 2g and h). In addition, owing to biocompatibility, hydrogel structures can be used, for example, in drug delivery [53, 54]. Encapsulated chemical substances can be released when the surrounding conditions, such as pH, vary [55], [56], [57].

## 3 Principles of shape transformation in 4D printing

Many studies on 4D printing involve shape transformation from a 2D planar structure to a 3D structure (Figure 1a). Because bending is indispensable for such 3D shape morphing, many 4D printing studies have employed shape-changing mechanisms based on the bending of layered structures [58], [59], [60], [61], [62]. As the simplest example of a layered structure, bilayer structures have been widely studied (Figure 1c and d), which are conceptually similar to bi-metal structures that respond to temperature changes. Bi-metal structures can be bent toward one side because of the difference in thermal expansion between two adjacent metal layers. Likewise, 4D-printed bilayer structures can be bent because of the strain difference between the two layers. The bending behavior can be analytically investigated using Timoshenko beam theory [63].

$$r=\frac{\text{t}\cdot \left[3\cdot {\left(1+m\right)}^{2}+\left(1+m\cdot n\right)\cdot \left(m^{2}+\frac{1}{m\cdot n}\right)\right]}{6\cdot {\left(1+m\right)}^{2}\cdot \epsilon }$$

where *r* is the radius of bending curvature, *t* is the total thickness of the beam (*t* = *t*_1_ + *t*_2_), *m* is the thickness ratio, *n* is the modulus ratio, and *ε* represents the strain difference between the two layers.

Ge et al. fabricated a 4D-printed bending structure with SMP fibers and a rubbery matrix that responds to heat [64]. They analytically calculated the bending angle of the structure by considering the viscoelasticity of the polymer materials. The total deformation was decomposed into mechanical and thermal deformations. A nonlinear multi-branch model was used to explain the time-dependent shape fixing and recovery characteristics of SMPs. After extracting relevant fitting parameters from experiment, the following relation was used to describe the bending behavior. Note that curvature *κ* is defined in Figure 1c, where *L* is the length of the beam.

$$\kappa =\frac{-BN_{\text{t}}+AM_{\text{t}}}{AD-B^{2}}$$

where *A* is the extensional stiffness, *B* is the coupling stiffness, *D* is the bending stiffness, $N_{\text{t}}$ is the thermal force, and $M_{\text{t}}$ is the thermal moment.

Bending occurs when the directions of upper and lower strains are the same (Figure 1c). However, if they are not in the same direction, twisting occurs (Figure 1d). More complicated shape morphing can also be achieved in 4D printing by controlling the magnitude and direction of strains in specific regions of printed structures.

**Figure 3:**
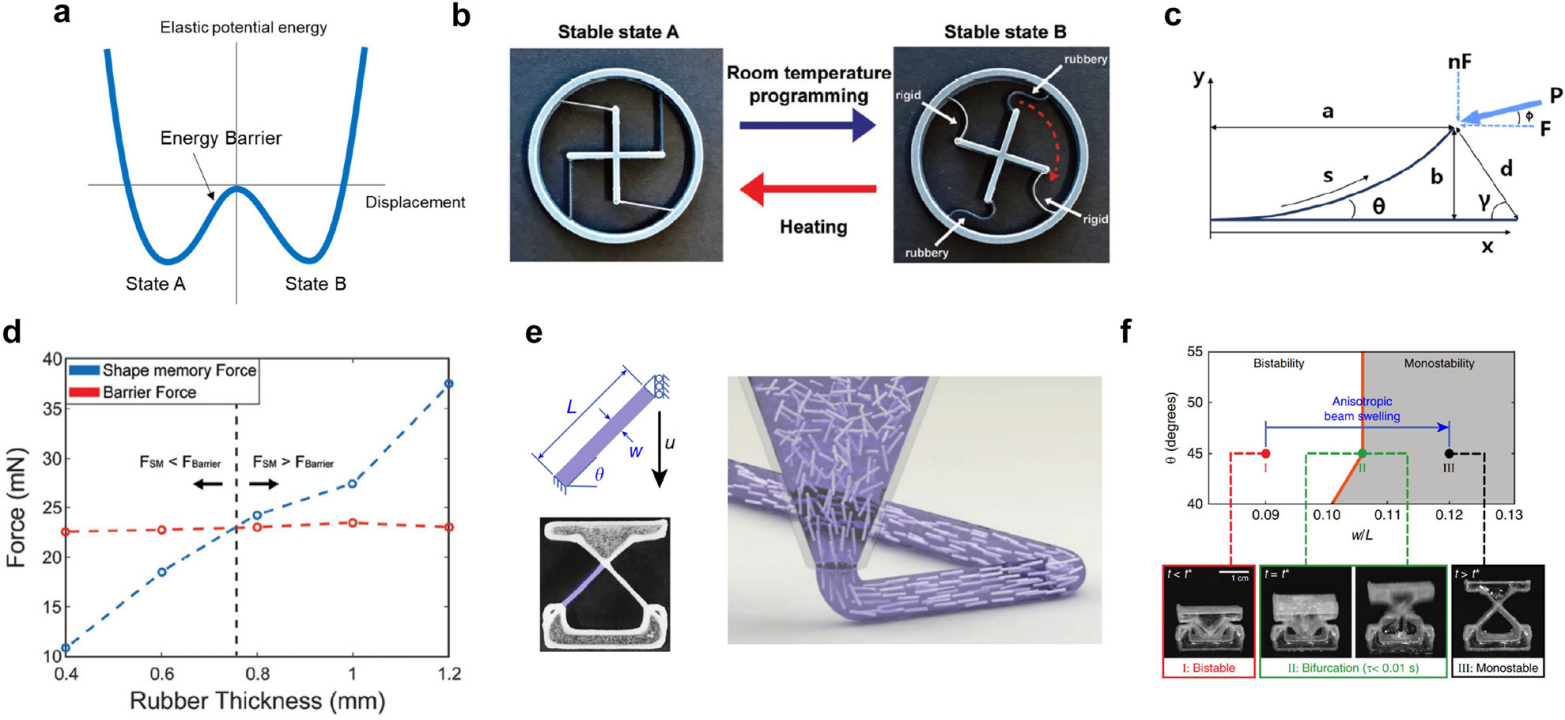
4D-printed bistable structures. (a) Elastic potential energy diagram of a bistable structure. (b) Thermal actuation of the rotational bistable structure. (c) Schematic for the reverse lateral deformation of the beam. (d) Comparison between the shape memory force of the rubbery SMP beams and the barrier force of the bistable structure. When the shape memory force exceeds the barrier force, the structure can be snapped into the original shape. Adapted from Ref. [74]. Copyright (2019) John Wiley & Sons. (e) A bistable structure printed with GF-PDMS. (f) Geometrical phase diagram of 4D-printed bistable structure. Because of anisotropic swelling of beams in toluene, an originally bistable structure becomes a monostable structure. Adapted from Ref. [76]. Copyright (2019) Springer Nature.

Another route for realizing highly reconfigurable structures is mechanical multistability. It allows for multiple stable configurations while reversible switching between them is possible – i.e., from a 3D structure to another stable 3D structure. Among the multi-stable structures, a bistable structure is the simplest case that has only two stable states in the potential energy diagram (Figure 3a). Two stable configurations (States A and B) are separated by an energy barrier, and sufficient force must be applied to the structure to overcome the barrier. Once passing the hill of the barrier, the bistable structure is automatically deformed into another stable configuration. Through this process, called snap-through, bistable structures can induce rapid, large-magnitude movement and can be used to simplify actuation without complicated control systems. They can also be used as mechanical switches because they do not require energy to maintain stable states. 3D printing of multistable structures can be realized in several ways: strained layers, compliant mechanisms, or mechanical metamaterials [65], [66], [67], [68]. 3D printing of multistable structures can enable highly reconfigurable components. By adopting smart materials into multi-stable structures, stimuli-responsive structures can also be obtained [69], [70], [71], [72], [73].

For example, Jeong et al. fabricated a multi-stable thermal actuator using PolyJet multi-material 3D printing [74]. Figure 3b shows a schematic of the rotational bistable structure. Two different SMPs (rigid and rubbery) were employed. The rigid beam had a fixed-pinned boundary whereas the rubbery beam had a fixed-fixed boundary. Two rigid beams define the overall bistability, whereas the rubbery beams act as a control knob. These multistable structures do not require heating in the programming stage, which significantly simplifies the actuation procedure (Figure 3b). At room temperature, the structure can be deformed to another stable state B. However, at elevated temperatures, the storage modulus of the rigid SMP beams significantly decreases. Then, the shape memory force of the rubbery SMP beams becomes a dominant factor in the potential energy of the structure, inducing transformation back to stable state A.

The bistable condition of the rigid SMP beams was analytically calculated from the beam theory, called the reverse-lateral (RL) condition. In Figure 3c, *a* and *b* define the *x* and *y* distances to the beam end, respectively. *s* indicates the tangential distance along the deformed beam, and *L* is the beam length. Then, the RL condition is expressed as [75]

$$\frac{d}{L}=\sqrt{{\left(1-\frac{a}{L}\right)}^{2}+{\left(\frac{b}{L}\right)}^{2}}\text{ and }\gamma =\frac{\pi }{2}-\tan^{-1}\left(\frac{1-a/L}{b/L}\right)$$

These two equations describe the stable position of the end of the beam. Once the angle of the load to the beam is determined, the deformed beam shape for bistability can be determined automatically. In their design, by varying the thickness of rubbery SMP beams, a balance between the energy barrier and shape-memory force was adjusted (Figure 3d), and this enabled controlled thermal actuation. The activation time for thermal actuation was also controlled; as the thickness of the rubber SMP increased, the activation time decreased.

Figure 3e shows another example of 4D-printed bistable structures [76]. A bistable structure with fixed and roller boundary conditions was fabricated by the DIW of glass fiber (GF) embedded polydimethylsiloxane (PDMS). The GF in a PDMS network can be aligned along the printing direction. The aligned GFs prevent PDMS from swelling along the aligned direction when the printed structure was immersed in toluene, and anisotropic swelling was achieved. The printed structure can have monostability or bistability upon geometrical parameters; there exists a certain slenderness ratio (*w*/*L*) that divides monostability and bistability (Figure 3f), which is called a bifurcation point. Because of the anisotropic swelling in toluene, the slenderness ratio of the GF-PDMS bistable structure can be increased, and bistability can turn into monostability as shown in Figure 3f. It was found that this transition occurred less than 0.01 s. Solvent logic operation was also demonstrated by combining a GF-PDMS structure (activated by toluene) with a GF-hydrogel structure (activated by water).

Multistability has also been considered for reconfigurable microelectronic devices. Fu et al. fabricated morphable 3D mesostructures using multistable structures based on pre-strained elastomers and 2D precursors [77]. Various examples were demonstrated for MOSFET, LED, and RF circuit integrated devices. Reversible reconfiguration was obtained by strategically organizing the time sequence of the strain release and by engineering the precursor designs.

More information on general 4D printing materials and mechanisms can be found in recent review articles [28], [29], [30].

## 4 Design and recent demonstration of remote light activation

### 4.1 Photochemical light activation

#### 4.1.1 General photochemical light activation

**Figure 4:**
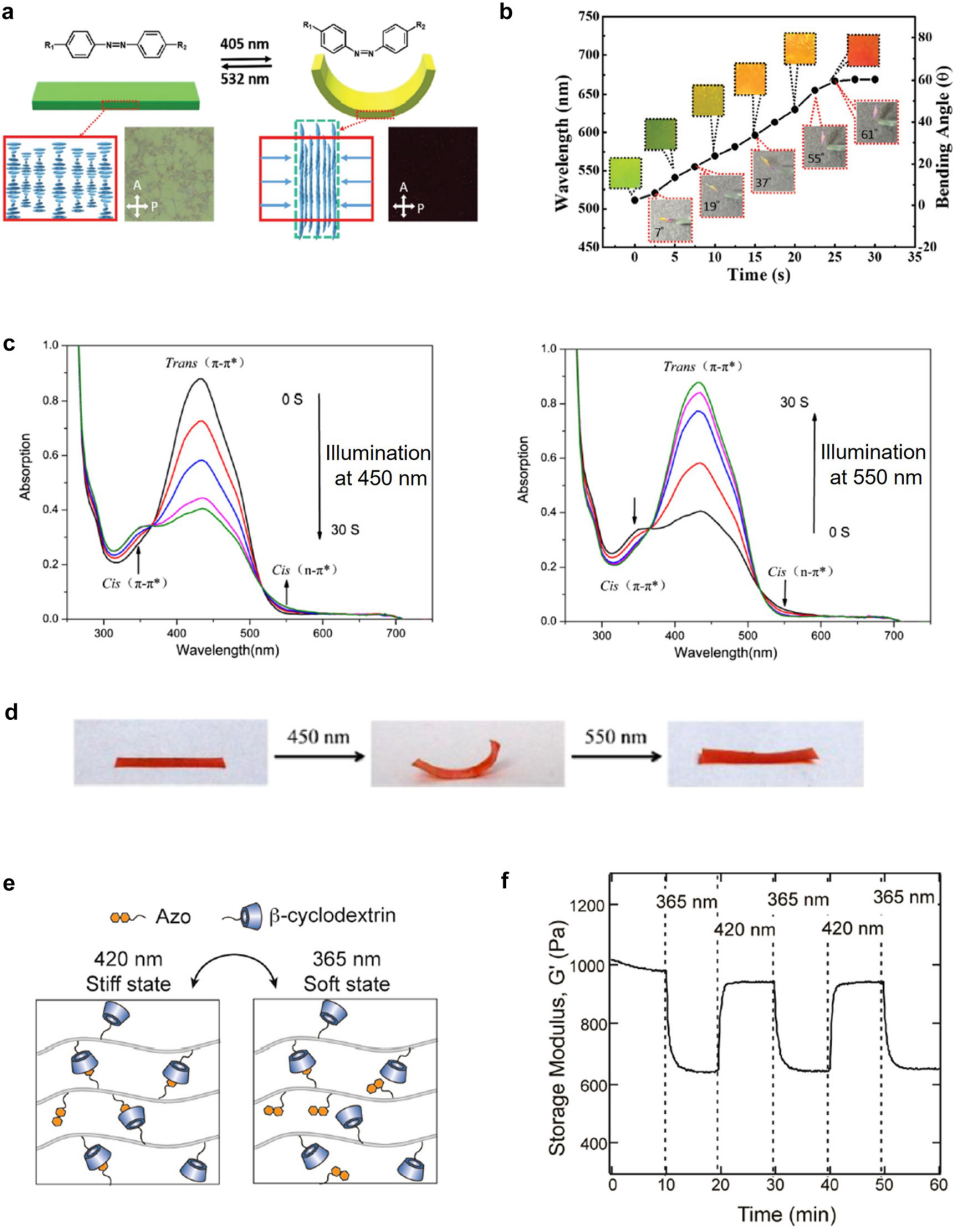
General photochemical structures based on azobenzene. *Light-activated LCE bending structure*: (a) schematic of a reversible bending structure. Helical LC structures can contract upon UV irradiation. The contraction near the top surface can induce bending. The original flat structure is recovered under 532 nm light illumination. Insets are the polarized optical microscopic images. (b) Bending behavior of the fabricated film, which also shows color changes due to the change of the helical pitch. Initially green film becomes red by UV light illumination. Adapted from Ref. [86]. Copyright (2020) Royal Society of Chemistry. *Light-activated SMP bending structure*: (c) UV–Vis absorption spectra of polyurethane/azobenzene dissolved in DMSO solution. Upon irradiation of UV light, the *trans* isomer gradually change its form to *cis*. This transition is reversed with visible light illumination. (d) Bending behavior of the polyurethane/azobenzene film. Adapted from Ref. [87]. Copyright (2017) ACS Publications. *Light activated hydrogel structure*: (e) schematic of isomerization of azobenzene. With 365 nm light irradiation, the crosslink density decreases (soft state). The transition is reversed with 420 nm light illumination (stiff state). (f) Reversible change of the storage modulus under the light illumination with alternative illumination wavelengths (365 nm, 420 nm). Adapted from Ref. [89]. Copyright (2018) ACS Publications.

Among photochemical reactive materials, azobenzene is the most widely studied for light-activated structures (Figure 4) [78], [79], [80], [81], [82], [83], [84], [85]. The molecular structure of azobenzene can be transformed from straight (*trans*) to bent (*cis*) upon UV light absorption. This *trans*-to-*cis* transformation is reversed upon visible light absorption (Figure 4a). By introducing azobenzene into the smart materials discussed in Section 2, it is possible to fabricate light-activated shape-morphing structures. The isomerization from *trans* to *cis* shortens the molecular chain of azobenzene. In this section, we introduce general photochemical design for light activation, which are fabricated by traditional methods, such as hot pressing and molding, and then we discuss the 3D printing of photochemical structures in the next section.

A light-activated structure mimicking chameleon’s skin was demonstrated by including azobenzene in cholesteric LCEs [86]. Cholesteric LCEs have a macromolecular helical structure (Figure 4a). This periodic helical structure plays the role of photonic crystals, similar to those in chameleon’s skin. By absorbing 405 nm light, the molecular configuration of azobenzene transforms from *trans* to *cis*. The overall volume contraction near the upper surface makes the LCE film bent toward the illumination source. At the same time, photoisomerization causes the helical pitch to change. As a result, the optical reflection peak of the LCE film spectrally shifts; therefore, the film’s color, which is originally green, changes to red. Figure 4b shows the bending behavior of the LCE film, which accompanies a color change from green to red.

A photochemical light-activated film was also demonstrated by including azobenzene groups in liquid crystalline polyurethane networks [87]. Polyurethane is a representative SMP material, and polyurethane films show shape-memory behavior after thermomechanical programming. Because of the liquid crystalline networks, the synthesized liquid crystalline polyurethane structures show a triple shape memory characteristic with two transition temperatures, *T*_g_ (44 °C) and *T*_iso_ (94 °C). The original shape was deformed at 105 °C and fixed in a first temporary shape at 64 °C. Next, it was re-deformed at 64 °C and re-fixed in a second temporary shape at 25 °C. After this thermomechanical programming, the structure returned to the first temporary shape at 64 °C and to its original shape at 105 °C. Figure 4c presents the UV-visible absorption spectra of polyurethane/azobenzene dissolved in a dimethyl sulfoxide (DMSO) solution. Under 450 nm light illumination, the absorption peak of the *trans* isomer gradually decreases because photo-isomerization occurs. When the polymer solution is illuminated by 550 nm light again, the *trans* isomer absorption peak gradually increases back. This demonstrates a reversible photochemical reaction in the polymer solution. Figure 4d shows the photo-induced bending and unbending behavior of the SMP film. After the film is stretched with thermomechanical programming, a fixed temporary shape can shrink by photo-isomerization. Due to the rigidity of the SMP polymer at room temperature, this behavior can be observed only above the *T*_g_ of the polymer. SMPs become rubbery above *T*_g_, and the illuminated upper region of the film can shrink. The difference in length between the upper and lower regions bent the structure toward the light source.

Hydrogels can also be light-activated by introducing azobenzene. Xiong et al. demonstrated a changeable modulus of hydrogels by the isomerization of azobenzene in supramolecular structures [88]. The isomerization of azobenzene from *trans* to *cis* states makes an originally crosslinked structure into a non-crosslinked structure. Therefore, the modulus decreases while the ductility increases (soft state). This phenomenon is a reversible process; the hydrogel/azobenzene recovers the crosslinked structure (stiff state) under 440 nm light illumination. Rosales et al. utilized a similar supramolecular hydrogel structure to control the release of a drug contained in a hydrogel structure by light [89]. When illuminated with 365 nm light, the hydrogel structure enters the soft state because of the decreased cross-linking density (Figure 4e). The structure can be returned to the stiff state by illuminating with 420 nm light. This transition between stiff and soft states can occur reversibly. In this way, a reversible modulus change of the hydrogel structure was demonstrated, which manifested an approximately 36% change in modulus (Figure 4f). The crosslinking density was correlated with the mesh size of the hydrogel structure. Proteins were encapsulated in the hydrogel structures, and light activation of drug release was demonstrated. The decrease in the crosslinking density by 365 nm light illumination led to an increase in drug release.

#### 4.1.2 Photochemical activation of 3D-printed structures

**Figure 5:**
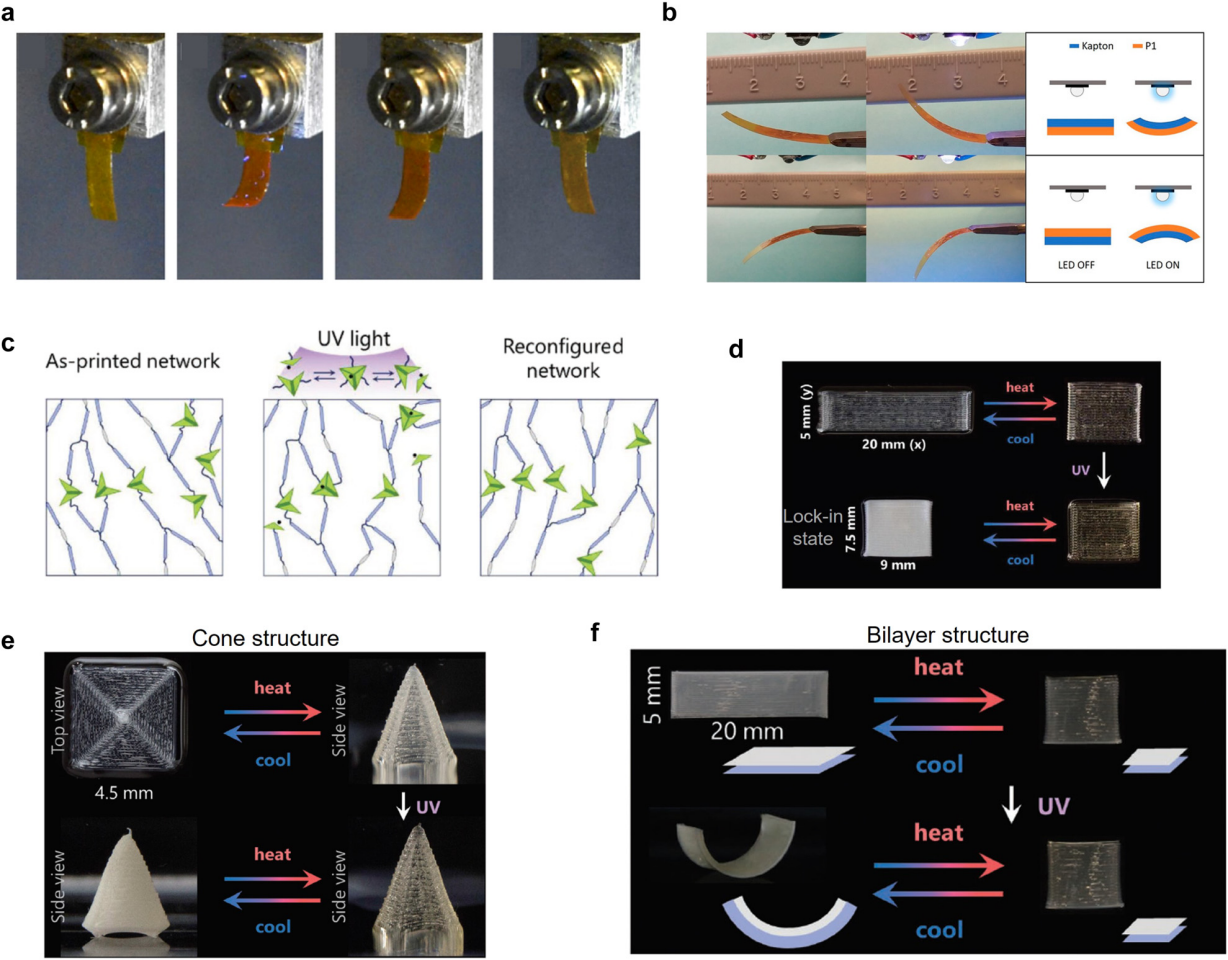
3D printing of photochemical structures. *DIW of LCE structures with azobenzene group*: (a) real images of LCE structure under UV and visible light illumination. Adapted from Ref. [90]. Copyright (2020) ACS Publications. *DIW of non-crosslinked LCs on a Kapton layer*: (b) bending behavior under light illumination. Bending occurs in 5 s and recovery occurs in 10 s. Adapted from Ref. [91]. Copyright (2018) ACS Publications. *DIW of ADT-included LCE structures*: (c) schematic of an LCE network showing the reconfiguration due to the covalent bonding exchange under UV light illumination. (d) Images of printed LCE strip actuation. UV illumination at 125 °C makes the structure to be locked in. The locked-in structure cannot recover the initial state. Instead, its opacity can change under a temperature change. (e) Actuation of a concentric square. It can be morphed to a square cone at high temperature. After UV treatment, it becomes opaque at ambient temperature. (f) Actuation of a bilayer structure. It is composed of ADT-contained upper and ADT-free lower layers. Before UV illumination, the bilayer structure can be actuated similar to a conventional single layer strip. After UV illumination, the lock-in of the upper layer makes the whole structure bent at ambient temperature. Adapted from Ref. [92]. Copyright (2020) John Wiley & Sons.

Recently, the 3D printing of various photochemical structures was demonstrated. For example, DIW was used to 3D print an LCE film that contains azobenzene for light activation [90]. Photopolymerizable inks were prepared, and the LCE mesogens were aligned along the printing direction. The printed structure was polymerized using 530 nm light illumination. Because of the LC alignment, the 3D-printed LCE film shrinks when heated to 100 °C. Azobenzene in the printed film was activated by illumination with 365 nm UV light causing a *trans*-to-*cis* transformation. Figure 5a shows the bending and unbending behavior when illuminated by light. The 3D-printed film took 4 min to bend under UV light illumination (50 mW/cm^2^) (second panel in Figure 5a). Ceasing the illumination, a slight stress relaxation occurred in the film (third panel in Figure 5a). The stress relaxation was caused either by a temperature gradient of the upper and lower parts of the film or by thermally induced isomerization from *cis* to *trans* states. The remnant portion of the *cis* isomers held the structure in the bent shape. After illumination with 455 nm light (4 mW/cm^2^) for 30 min, the film recovered its initial state.

Light-activated bilayer actuators have also been fabricated using non-crosslinked LC polymers [91]. Unlike conventional LCE structures, non-crosslinked LC polymers were dissolved in an organic solvent and printed using DIW. Azobenzene was attached to the backbone of the linear LC monomers. Non-crosslinked LC polymers were printed on a passive Kapton substrate to form a bilayer structure (Figure 5b). Because the *cis* state of the azobenzene group increases the volume of the structure in this case, bending occurs toward the Kapton layer side. Figure 5b shows the bending behavior of the bilayer film for the two different orientations of the bilayer; in either case, bending occurs toward the Kapton layer side. Bending can occur within 5 s after a UV source is turned on. When the UV source is turned off, the film takes only 10 s to recover its initial flat structure because of the thermal relaxation from the *cis* to *trans* state.

It is also possible to fix the shape of reconfigured structures. Davidson et al. reported 3D-printed LCE structures using DIW, which exhibited light-induced *lock-in* behavior [92]. Instead of azobenzene, allyl dithiol (ADT) was introduced into the LCE structures. ADT can dynamically exchange bonding upon UV light absorption (Figure 5c). 3D-printed LCE inks containing ADT were photopolymerized with green light. Owing to the alignment of the LC mesogens during printing, the photopolymerized LCEs had an aligned smectic state. When heated above 90 °C, the printed film shrank and recovered its original length when cooled (Figure 5d). The contraction and recovery were fully reversible for at least 20 cycles. When the film is illuminated by UV light and heated to 125 °C, the ADT exchanges its bonds. Dynamic bond exchange disturbs the alignment of the LCE. When cooled back to room temperature, the film became opaque (Figure 5d), and it could not recover its initial state. This state is termed the *lock-in* state. Diverse active structures can be fabricated by varying the printing path. Figure 5e shows a concentric square that reversibly transforms to a square cone, which is locked-in after UV illumination. Figure 5f shows a bilayer structure consisting of an active layer containing ADT and a passive layer without ADT. By heat treatment, the bilayer structure can only be actuated in the plane. However, after UV illumination, the upper active layer with ADT was locked-in. Only the lower passive layer can recover its length. The length difference between the upper and lower layers bends the entire structure.

### 4.2 General photothermal light activation

#### 4.2.1 General photothermal activation via dyes and colored materials

Photothermal heating is another common method used for remote light activation. Remote light activation can be realized by mixing dyes or nanomaterials (e.g., carbon-based materials, metal nanoparticles, etc.) in a smart material matrix such as SMP, LCE, or hydrogel. In Section 4.2, we first describe general photothermal structures based on nanomaterial composites, which are fabricated by traditional patterning. Then, we discuss how such light-activation mechanisms are employed for 3D-printed active structures reported in the recent literature (Sections 4.3–4.5).

Dye molecules can selectively absorb light in a certain wavelength range. Absorbed light can be converted to heat, leading to an increase in the temperature. Using this photothermal mechanism, heat-responsive smart materials can be actuated by light. For example, Rogóż et al. fabricated a millimeter-scale crawling snail robot using dye-doped LCEs [93]. In their work, glycerin played a role similar to that of the mucus of snails. The fabricated LCE strip underwent repetitive contraction and recovery by laser scanning for light-driven locomotion. Wani et al. fabricated a light-driven artificial flytrap by incorporating dye molecules into splay-aligned LCEs [94]. The light-activated gripper was realized by attaching an LCE strip to an optical fiber tip. Zeng et al. fabricated an LCE iris structure by incorporated dyes. The initially open iris was closed by itself upon light illumination [95]. Shahsavan et al. demonstrated a bioinspired underwater soft robot by incorporating photothermal dye molecules. The fabricated soft robot can walk, crawl, jump, and even swim in water with proper laser irradiation [96].

**Figure 6:**
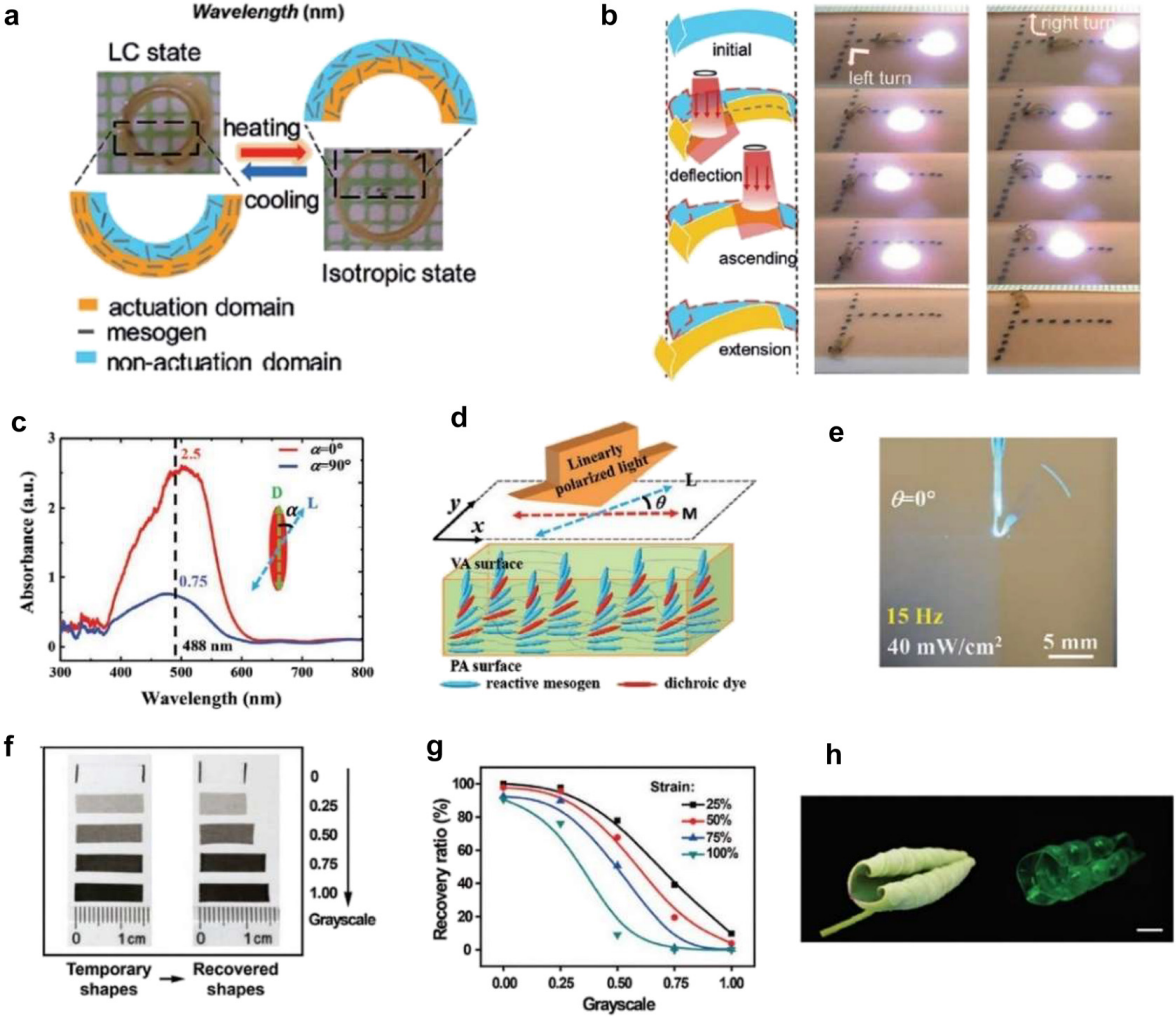
General photothermal method for light activation: dye molecules and colored materials. *NIR activated bilayer LCE structure*: (a) schematic of a bilayer LCE structure. NIR dyes are included in an actuation domain. The contraction of the actuation domain can make the structure flip under the NIR irradiation. (b) Locomotion of the fabricated crawler. The first column is a schematic of the crawler actuation under NIR laser scanning. The second column shows the crawler turning left, while the third column shows the crawler tuning right. Adapted from Ref. [97]. Copyright (2018) John Wiley & Sons. *Polarization-dependent morphing of LCE structures*: (c) absorption spectra of a dichroic dye. α is the angle between the main axis of the dichroic dye and the polarization of light. (d) Schematic of the LCE structure with splay orientation. The parallel alignment (PA) surface is aligned along the main axis of the film, while LC mesogens are aligned vertically on the vertical alignment (VA) surface. *θ* is the angle between the PA alignment direction and the polarization of the light. (e) Image of the oscillating film under the light polarization of *θ* = 0°. The film oscillates at 15 Hz. Adapted from Ref. [98]. Copyright (2021) John Wiley & Sons. *Light-programmable SMP structures with grayscale printing*: (f) images showing the different recovery capacity for varying grayscales. (g) Recovery ratio as a function of the grayscale. (h) Images of a biomimetic leaf actuated by visible light illumination (scale bar: 5 mm). Adapted from Ref. [104]. Copyright (2020) John Wiley & Sons.

Ge et al. fabricated a dye-doped LCE bilayer actuator using near-infrared (NIR) absorbing dyes (Figure 6a) [97]. Before crosslinking the LCE strip, it was uniaxially stretched by 330% to align the mesogens. Then, only one side of the LCE bilayer strip was crosslinked in the aligned state with UV light exposure. After crosslinking, the LCE strip was placed in hot water for stress relaxation while the other un-crosslinked side was kept isotropic. In this step, the fabricated strip curled toward the non-crosslinked side. The isotropic un-crosslinked side of the LCE strip was crosslinked with UV light exposure at room temperature. The fabricated LCE strip had a bilayer structure with actuation and non-actuation domains (Figure 6a). The length of the actuation domain can be shortened at high temperatures, leading to flipping of the curved bilayer. Using proper NIR laser irradiation, locomotion and directional changes were demonstrated; the bilayer actuator could switch the moving direction to either left or right under proper laser guidance (Figure 6b). Crawling on a 15° slope was also demonstrated.

The polarization of light can be another control knob for shape morphing. For example, polarization-dependent LCE actuators were demonstrated by introducing dichroic dyes (Figure 6c–e) [98]. The dichroic dye RL202, which has an absorption peak around the wavelength of 488 nm, shows polarization-dependent absorption; light absorption is 3.4 times stronger at 488 nm along the orientation of dichroic dyes, compared to that of the perpendicular orientation (Figure 6c). The LCE film has a *splay* configuration, where mesogens are aligned in the parallel direction along the long axis at one surface while aligned in the perpendicular direction at the other surface – called parallel alignment (PA) and vertical alignment (VA) surfaces (Figure 6d). Here, *θ* is defined as the angle between the polarization direction of the incident light and the alignment direction of mesogens at the PA surface. For the polarization of *θ* = 0°, the PA surface has more shrinkage than the VA surface; this difference leads to the bending of the LCE film. Owing to the splay configuration, the bending angle depends on the polarization angle *θ*. The bending angle reaches a maximum at *θ* = 0° and decreases as *θ* increases.

A polarization-dependent oscillator was demonstrated using the same LCE film (Figure 6e). A threshold laser power exists above which the LCE film can oscillate. The mechanism of this light-driven oscillation can be described as follows: (i) during oscillation, the bent shape of the LCE film deviates from the optimal alignment angle between the polarization angle of light and the direction of the dichroic dye molecules, (ii) the decrease in temperature makes the LCE film recover its shape slightly, and (iii) the re-oriented dichroic dye molecules begin to absorb more light and cause the structure to be bent again. These steps are repeated very quickly, which causes the LCE film to oscillate. At a laser power of 40 mW/cm^2^, the oscillation frequency reached 15 Hz at *θ* = 0°.

The coating or printing of colored materials can also induce light activation [99], [100], [101], [102]. Li et al. coated polydopamine onto planar SMP sheets [103]. Owing to the polydopamine coating, NIR light energy was converted to thermal energy, and the SMP sheets was actuated. In addition, polydopamine was erasable using NaOH solution. Pre-strained SMP sheets with inkjet-printed patterns can be programmed by light too. For example, shape morphing was demonstrated using poly(L-lactide) (PLLA) as an SMP material [104]. PLLA has crystalline domains at a certain temperature between *T*_g_ and the melting temperature. Thermomechanically stretched SMP films with inkjet-printed patterns show different recovery capacities after light illumination, depending on the grayscale patterns. Figure 6f and g compare the recovery capacity of different grayscale samples. As the grayscale is higher, the PLLA reaches a higher temperature under light illumination. This leads to a more crystalline film, which, in turn, disturbs the recovery of SMPs. Using this feature, different structural morphings were realized depending on the grayscale pattern and light illumination. For example, the shape morphing of a bioinspired leaf was demonstrated (Figure 6h).

#### 4.2.2 General photothermal activation via carbon-based materials

Carbon-based nanomaterials such as carbon nanotube (CNT), carbon black (CB), and graphene oxide (GO) can absorb light over a wide frequency range (from optical to microwave frequency region) and can be used for photothermal activation [105], [106], [107]. For example, Li et al. introduced single-walled CNTs (SWCNTs) into LCE structures to utilize them in a solar cell system [108]. Inspired by *heliotropism*, they placed a solar cell panel on three pillars composed of SWCNT-embedded LCEs. The contraction of LCEs made a solar cell panel directing toward the Sun. Cheng et al. fabricated NIR-Vis-UV responsive actuator films by introducing GOs in an LCE matrix [109]. GOs can absorb NIR-Vis light and convert incident light energy to heat. Dye molecules were also included to absorb UV light. In this manner, they demonstrated actuating-film structures under natural sunlight.

**Figure 7:**
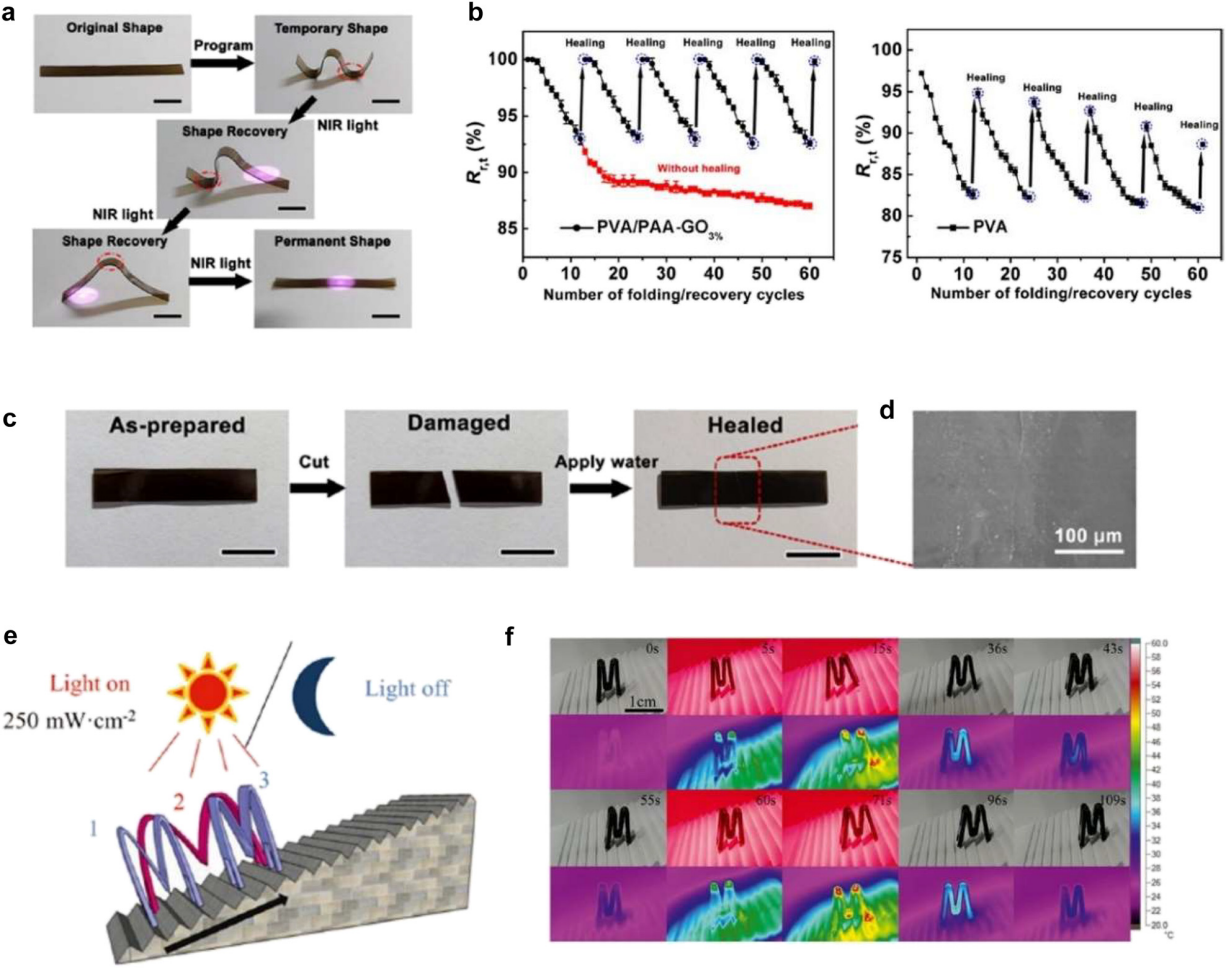
General photothermal method for light activation: carbon-based materials. *Self-healable SMP structures activated by NIR light*: (a) images of shape memory processes under NIR light illumination. GO effectively absorbs NIR light and produces heat. (b) Recovery ratio of PVA/PAA-GO_3%_ with or without healing. (c) Images of the healing process. The fractured part is completely repaired by water. (d) SEM image of the healed film. Adapted from Ref. [110]. Copyright (2019) ACS Publications. *Reversible shape memory crawling robot*: (e) schematic of the crawling robot locomotion under sequential light switching. (f) Optical and IR images of the crawling robot ascending stairs by sequential light switching. Adapted from Ref. [111]. Copyright (2019) ACS Publications.

“Healable” SMP films were also fabricated, which were responsive to NIR light by introducing GOs [110]. 3 wt% of poly(acrylic acid) (PAA) grafted GO nanosheets were introduced into a poly(vinyl alcohol) (PVA) matrix. The fabricated SMP film structure was programmed into a temporary W shape (Figure 7a). Upon NIR light illumination, the SMP film recovered its original shape within a few seconds. However, this recovery mechanism suffers from gradual degradation over recovery cycles (red curve in Figure 7b). Degraded SMP films can be *healed* by water because of reversible hydrogen bonding between PAA and PVA (black curve in the left panel of Figure 7b, PAA/PVA-GO_3%_). In contrast, the PVA control sample still shows gradual degradation even after healing (right panel of Figure 7b, PVA). Furthermore, mechanically damaged structures can be repaired completely. Figure 7c shows a healing feature of PAA/PVA-GO_3%_. The damaged sample was healed by water. Figure 7d shows the SEM image of the originally fractured boundary in the healed sample.

The photo-thermal approach was used to develop a light-driven crawling robot using carbon-based materials [111]. Ball milling and hot pressing were used to mix CNTs with poly(ethylene-co-octene) (POE). Unlike standard SMPs, POE shows repeated shape morphing owing to the broad melting temperature regime of semicrystalline structures. Inspired by a leopard, the crawling robot was demonstrated by deforming the strip into an M shape (Figure 7e). The folded legs of the M-shape structure can be partially recovered and stretched upon IR irradiation. When the light is turned off, cooling down the structure makes legs contract back. Crawling movement was demonstrated by turning the light on and off repeatedly (Figure 7f). Finely-dispersed CNT/POE materials possess effective electrical paths. The electrical actuation of a gripper was also demonstrated at 36 V.

Shape morphing structures under microwave radiation have also been reported. Patel et al. introduced multi-wall CNTs (MWCNTs) into a polyurethane matrix [112]. A deformed structure, which was initially coil-shaped, can recover its original shape in a microwave oven. As the weight percentage of MWCNTs increases, an increase of shape fixity and modulus was reported.

#### 4.2.3 General photothermal activation via metal nanoparticles

**Figure 8:**
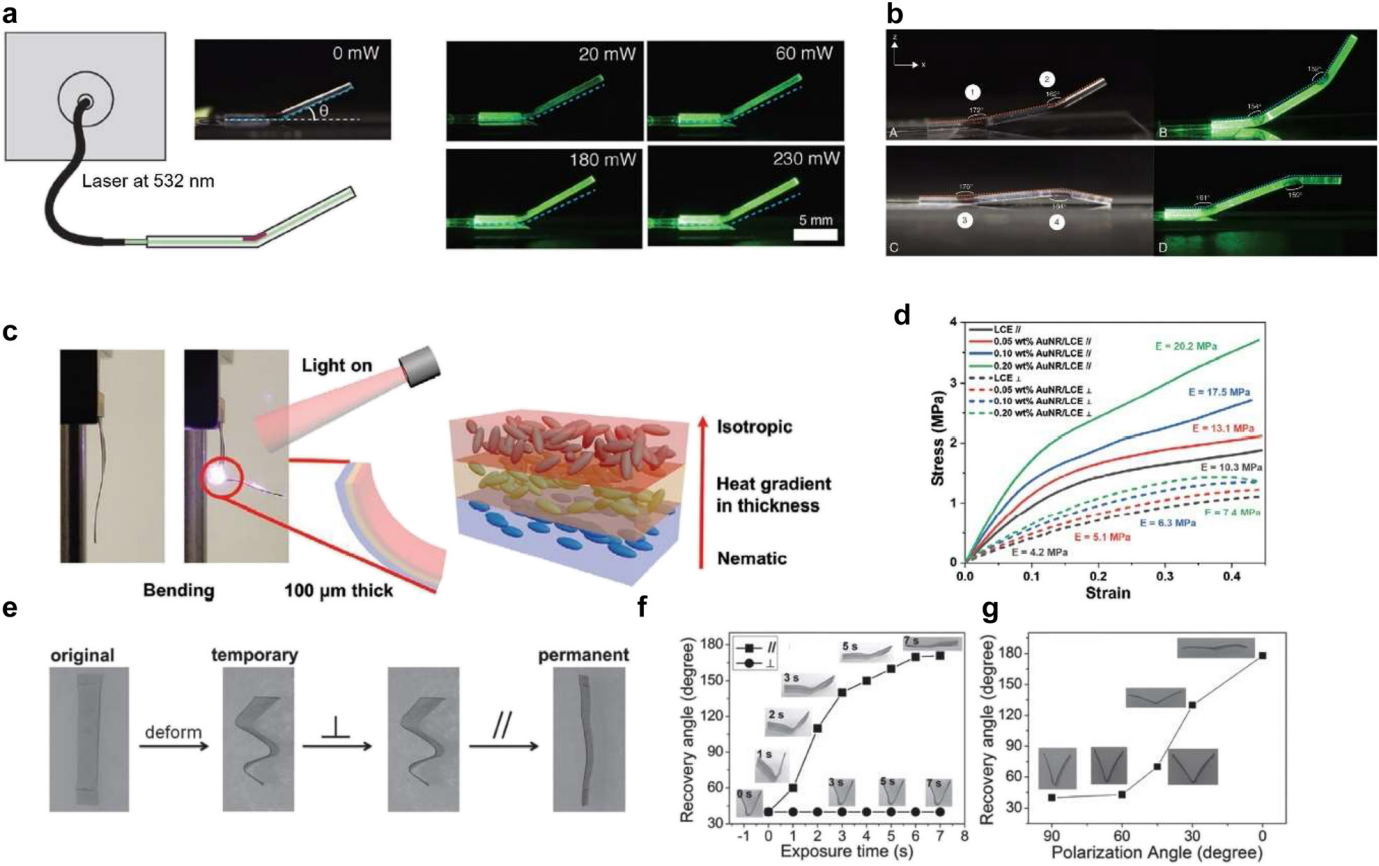
General photothermal method for light activation: metal nanoparticles. *Light-activated LCE waveguide*: (a) images of the actuating LCE waveguide with various laser power. The dashed line indicates the original position of the waveguide. (b) Images of LCE waveguides having two actuating hinges. Adapted from Ref. [113]. Copyright (2019) John Wiley & Sons. *Light-activated LCE bending structure*: (c) images of bending under light illumination. The upper layer of the LCE film transforms to the isotropic state that induces bending. (d) Stress-strain curve with different wt% of AuNRs and directions. Adapted from Ref. [115]. Copyright (2020) John Wiley & Sons. *Polarization-dependent shape memory effect*: (e) images of recovery characteristics of the SMP strip. (f) Recovery angle of the strip versus the exposure time for vertical and parallel alignments to the polarization of incident light. (g) Recovery angle of different polarization angles. Adapted from Ref. [116]. Copyright (2013) John Wiley & Sons.

Various photothermal shape morphing structures were demonstrated by introducing metal nanoparticles into LCEs or SMPs. Metal nanoparticles support surface plasmon resonances and can induce strong light absorption. An LCE waveguide actuator, for example, was demonstrated by encasing an Au-containing precursor on the LCE waveguide and precipitating gold nanoparticles (AuNPs) with UV light illumination (Figure 8a) [113]. When 532 nm laser light is coupled to the LCE waveguide, an actuating hinge in the waveguide can be bent toward the AuNP side within a few seconds while light is guided through the waveguide. Figure 8a shows the bending of the LCE waveguide at various laser power. The waveguide was bent more for higher laser power; at 230 mW, the waveguide was bent about 13°. LCE waveguides with two actuating hinges were also demonstrated (Figure 8b). Depending on the hinge location (either top or bottom), the waveguide transformed into different shapes.

Ding et al. fabricated light-induced actuating nanotransducers by coating AuNPs with poly(N-isopropyl acrylamide) (PNIPAm) [114]. A swelling degree of PNIPAM can be controlled in water with temperature. At room temperature, PNIPAM-coated AuNPs were separated by certain distances because of swelled PNIPAM. Laser illumination induced the plasmonic heating of AuNPs; then, the deswelling of coated PNIPAM occurred, and nanoparticles became agglomerated. After cooling, the swelling of PNIPAM made the agglomerated particles apart again.

Surface plasmon resonances in Au nanorods (GNRs) can cause significant photothermal heating. Additionally, they show polarization-dependent behavior because of their elongated shape. For example, AuNRs were added to an LCE matrix for shape morphing [115]. AuNRs with poly(ethylene glycol) (PEG) ligands were infiltrated during the polymerization of the LCE. Well-dispersed PEG-AuNRs could be aligned along the LC oligomers due to capillary force during the infiltration. The fabricated LCE film showed bending when an 800 nm light source illuminated the sample in 5 s (Figure 8c). By introducing AuNRs up to 0.2 wt%, a modulus of the LCE film was improved. Figure 8d compares moduli for different wt% of AuNRs and the LC direction. The pristine LCE film has a modulus of 10.3 MPa, while the LCE film containing AuNRs has a modulus of 20.2 MPa along the aligned axis.

Au nanoparticles have also been introduced into SMP matrices [116, 117]. Zhang et al. demonstrated the SMP composite films that exhibit polarization-dependent recovery behavior (Figure 8e). Thermomechanically deformed SMP strips were recovered under NIR light illumination. When the polarization of incident light was perpendicular to the AuNR direction, no shape recovery occurred. However, a full recovery of the deformed film occurred when the polarization is in parallel to the AuNR direction. Figure 8f compares the recovery of the deformed SMP film for two polarizations. After 7 s of illumination, the parallel polarization showed full recovery while the perpendicular polarization did not show recovery at all. Figure 8g shows how recovery is affected by the polarization angle; as the polarization of incident light is aligned to the AuNR direction, more recovery was obtained. Again, the parallel polarization (0°) shows the full recovery of the deformed SMP film.

#### 4.2.4 General photothermal activation via hybrid structures of metal nanoparticles and carbon materials

**Figure 9:**
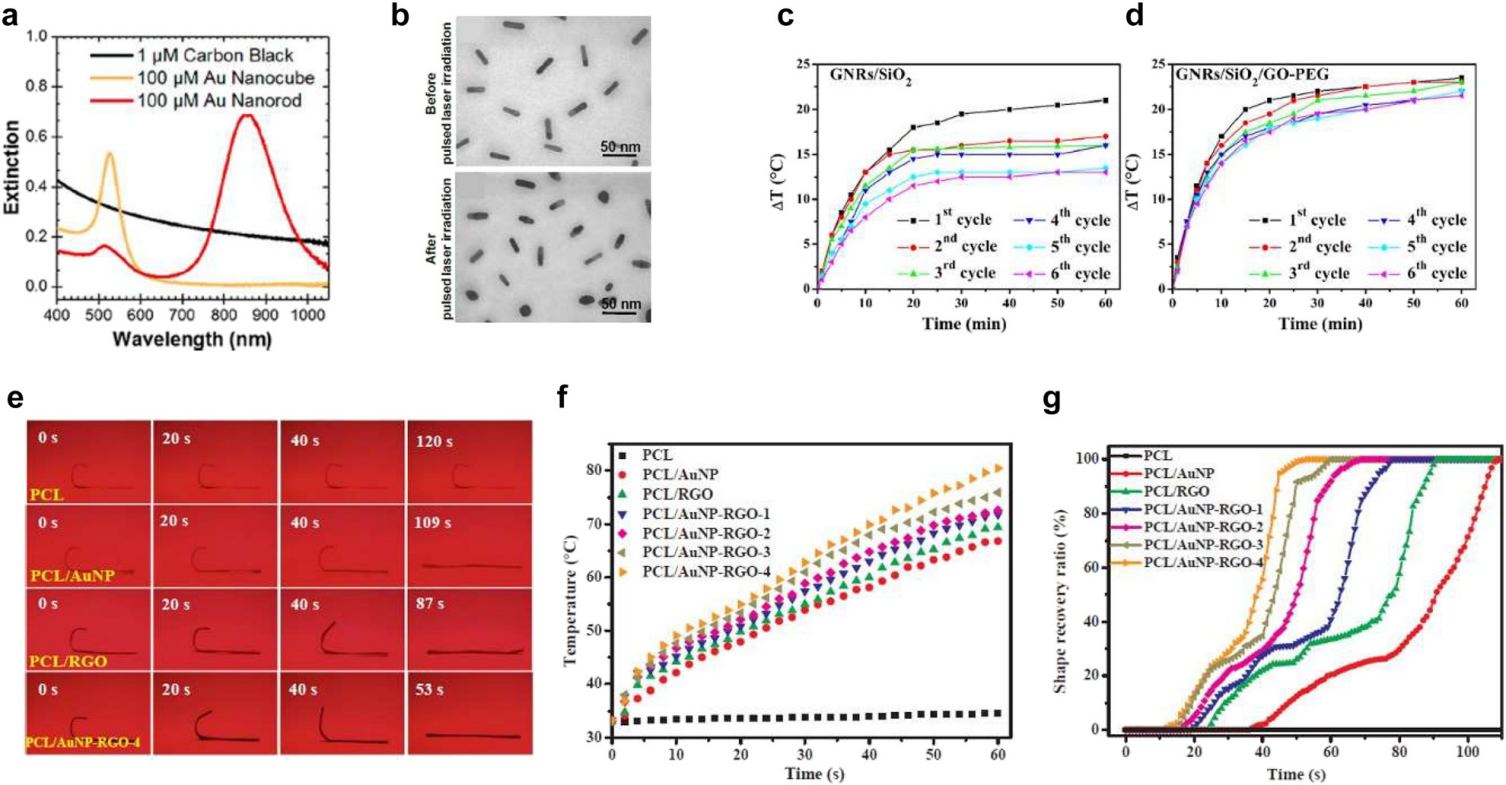
General photothermal method for light activation: hybrid of metal nanoparticles and carbon nanomaterials. (a) Extinction spectra of carbon black, gold nanocubes, and gold nanorods. Gold nanoparticles have distinct extinction peaks, while carbon black shows a broad extinction spectrum. Adapted from Ref. [118]. Copyright (2018) ACS Publications. (b) TEM images of gold nanorods before and after laser irradiation. Damage occurs after laser irradiation. Adapted from Ref. [119]. Copyright (2010) OSA Publishing. (c) Temperature increase of GNRs/SiO_2_ under laser irradiation. (d) Temperature increase of GNRs/SiO_2_/GO-PEG under laser irradiation. It shows a better thermal stability. Adapted from Ref. [124]. Copyright (2019) Elsevier. (e) Images of SMP composites showing shape memory behavior under NIR light illumination. (f) Temperature increase as a function of time under NIR light illumination. (g) Shape recovery ratio as a function of time under NIR light illumination. RGO and AuNP have synergetic effects. Adapted from Ref. [125]. Copyright (2019) Elsevier.

Carbon-based materials can absorb light in a wide frequency range. Furthermore, their high thermal conductivity provides rapid heat transfer. On the other hand, metal nanoparticles supporting surface plasmon resonances can absorb light more selectively in a certain wavelength range. The absorption wavelength can be engineered by tuning the size or shape of metal nanoparticles. AuNPs are known to have excellent photothermal energy conversion. Figure 9a shows the UV-visible extinction spectra of CB, Au nanocube, and GNR [118]. CB shows a broad absorption spectrum (black curve), while Au nanocube and GNR exhibit selective extinction peaks (yellow and red curves). However, Au nanoparticles suffer from gradual degradation after a few cycles of heating-up and cooling-down (Figure 9b) and have a drawback in thermal stability [119].

To solve this problem, a hybrid structure of Au nanoparticles and carbon-based nanomaterials was considered [120], [121], [122], [123]. For example, GO-capped GNR structures were fabricated for drug delivery [124]. First, GNRs/mesoporous silica nanoparticles were fabricated and capped by GO-PEG. Figure 9c and d compares temperature changes from the first-to-sixth cycle of GNRs/SiO_2_ and GNRs/SiO_2_/GO-PEG, respectively. GNRs/SiO_2_ (i.e., without carbon materials) shows a gradual deterioration of thermal stability through sequential cycles, as indicated by decreased temperature increases upon light illumination. Meanwhile, GNRs/SiO_2_/GO-PEG (i.e., with carbon materials) shows higher thermal stability than GNRs/SiO_2_.

A hybrid structure of AuNPs and reduced GO (RGO) was also introduced into SMPs [125]. AuNP-RGO nanohybrids were mixed in a poly(ε-caprolactone) (PCL) solution and hot-pressed. Figure 9e presents the shape-memory behavior of SMPs embedded with various nanomaterials. An 808 nm NIR laser was illuminated to thermomechanically programmed SMP strips. Pristine PCL did not show a shape-memory effect, while PCL/AuNP-RGO exhibited the fastest shape recovery under light illumination. Figure 9f shows temperature changes over time under 808 nm laser illumination. The AuNP-RGO hybrid structure showed *synergetic* effects on photothermal heating. Figure 9g shows the shape recovery ratio of SMP strips embedded with different nanomaterials. As the weight percentage of AuNPs increased in the hybrid structure, faster shape recovery responses were observed.

#### 4.2.5 General photothermal activation via semiconductor nanoparticles

Copper sulfide (CuS) is a semiconductor nanoparticle showing efficient photothermal conversion. Instead of surface plasmon resonances, the intrinsic energy band transition can make CuS efficiently absorb NIR light. Because of low cost and high photothermal efficiency, CuS nanoparticles have been intensively studied, for example, in cancer therapy [126], [127], [128]. Li et al. introduced CuS nanoparticles into an SMP (polyurethane) matrix [129]. By adding 0.2 wt% of CuS nanoparticles, a deformed polyurethane strip recovered its original shape in 21 s upon the illumination of 808 nm NIR light.

### 4.3 Photothermal activation of 3D-printed structures

#### 4.3.1 Photothermal activation of 3D-printed SMP structures

**Figure 10:**
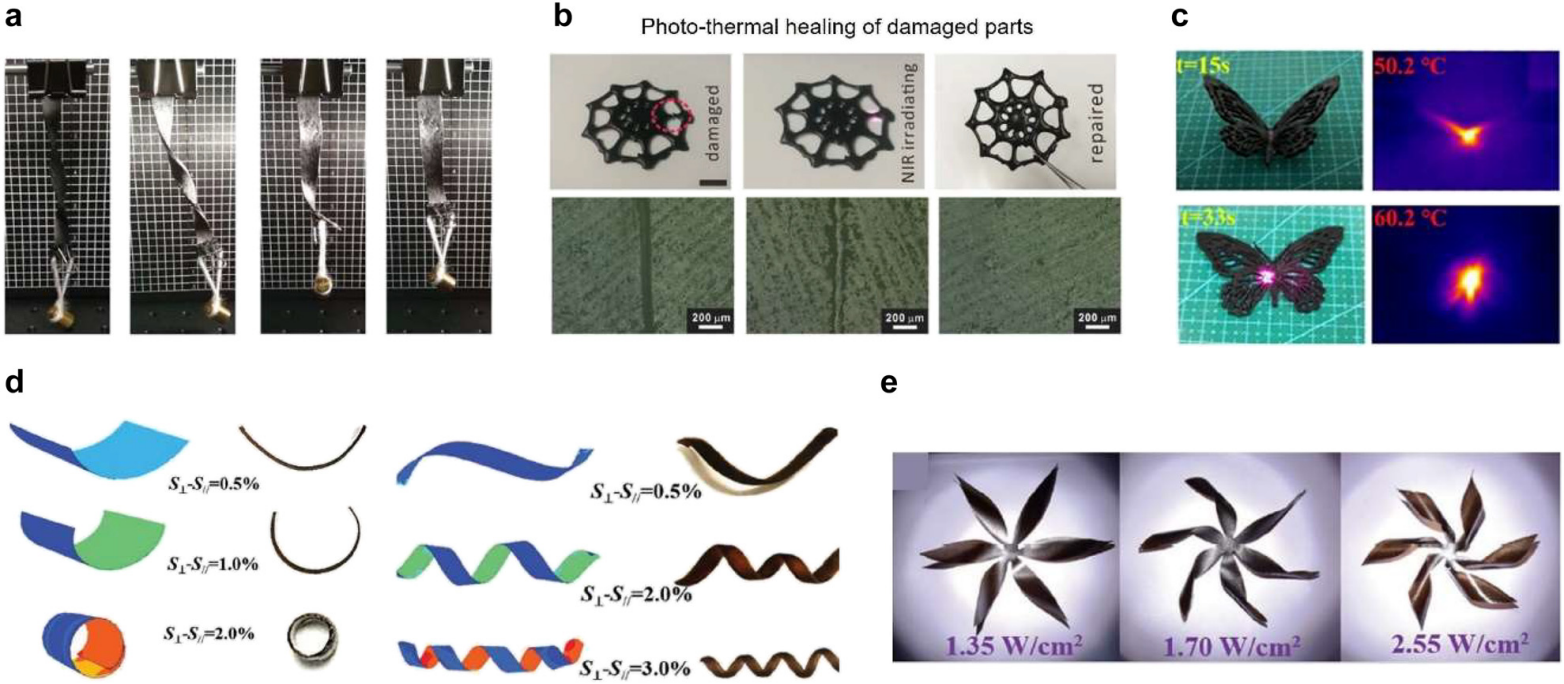
Photothermal activation of 3D-printed structures. *DIW of Light-activated self-healable SMP structures*: (a) images of a deformed strip recovering its shape under NIR light illumination. (b) Images of a damaged sample and the healing process using NIR laser irradiation. The SEM images of each step are also shown. Adapted from Ref. [130]. Copyright (2019) Royal Society of Chemistry. *FDM of NIR-activated SMP structure*: (c) optical and IR images of a butterfly shape, which shows shape recovery by NIR light. Adapted from Ref. [131]. Copyright (2020) Elsevier. *DIW of Multi-stimuli induced shape morphing in hydrogel structures*: (d) experimental and simulated bending and twisting for different strain values. (e) Images of flower structures under light illumination. Adapted from Ref. [135]. Copyright (2020) John Wiley & Sons.

More complicated structures can be created using 3D printing. Various 3D printing methods were recently considered for light-activated structures. For example, DIW was used to create self-healing, light-activated structures by integrating aniline trimer (AT) into a polyurethane matrix [130]. When temperature increases, the viscosity of the polyurethane matrix gradually decreases, permitting the DIW of the polyurethane matrix. This viscosity change is induced by thermally dynamic covalent bonding of the polyurethane matrix. Figure 10a shows the shape-memory characteristic of the printed strip. A twisted strip hanging a weight of 5 g showed shape recovery under NIR light illumination. Additionally, thermally dynamic covalent bonding can induce the self-healing of the polyurethane matrix. Figure 10b shows the pictures (upper panel) and microscope images (lower panel) of the printed patterns. It is demonstrated that damaged parts can be repaired under NIR laser irradiation.

Dopamine-modified MWCNTs were also introduced into a thermoplastic polyurethane network, where MWCNTs were coated with poly-dopamine (PDA) [131]. Both PDA and MWCNT show significant absorption in the NIR region. The thermal conductive paths in the polyurethane matrix are increased due to the additional hydrogen bonding between PDAs. Therefore, the polyurethane film including PDA-coated MWCNTs have nearly twice higher thermal conductivity. Furthermore, the mechanical properties such as elongation modulus, tensile strength, and elongation-at-break are enhanced, too. Complex structures were fabricated using FDM 3D printing, and shape-memory effects were demonstrated. Figure 10c shows the pictures and thermal images of the printed butterfly structure. The deformed structures were restored to their original, flat state using NIR illumination.

Cui et al. demonstrated the shape-memory behavior of a brain structure under NIR illumination by incorporating graphene into an SMP matrix [132]. Improved cell differentiation was demonstrated, which was attributed to the optoelectronic characteristics of graphene. Keneth et al. fabricated sequential shape morphing structures using the multi-material DLP technique [133]. They incorporated CNTs into an SMP matrix. External and internal lids in a printed box were sequentially opened under the illumination of a 395 nm LED source.

#### 4.3.2 Photothermal activation of 3D-printed hydrogel structures

3D-printed hydrogel structures were also used for photothermal light activation. Han et al. used SLA to fabricate cephalopod-inspired light-activated chromatophores (LACs) [134]. By incorporating PDA nanoparticles (PDA-NPs) into a thermo-sensitive hydrogel matrix, 3D-printed structures were actuated upon illumination with white light (7.7 mW/cm^2^). They fabricated LACs by printing three different materials. Combining a rigid material for frames and a stretchable material with PDA-NPs-doped hydrogel for muscles, they could fabricate reversibly actuating LACs in water.

Shape-morphing hydrogel structures were also fabricated via DIW, which responded to multiple stimuli such as water, heat, and light [135]. Graphene oxide (GO) flakes were introduced into a sodium alginate (SA) matrix. During the 3D printing, GO flakes were aligned to the printing direction because of the shear force and gravity. Aligned GO flakes produced anisotropic features to the printed structure. Utilizing the bilayer bending mechanism, the fabricate structure could be bent to 0° and 90° printing directions and could be twisted along 45° and −45° printing directions under multiple stimuli. Vapor makes the structure to expand anisotropically. On the other hand, heat and light can make the structure to contract. Figure 10d shows the simulation (left) and sample images (right) of the bending and twisting structures in water for various strain differences. The larger the strain difference was, the more deformation occurred. A flower structure was printed using these features for a shape-morphing demonstration. Figure 10e shows the images of the printed flower, which is actuating under light illumination. Originally flat structures could be morphed into 3D flower structures as petals were twisted under xenon lamp illumination. The deformed structure can be recovered to the original flat shape when water vapor is introduced. This shape morphing happens in a few seconds and occurs reversibly for more than 100 cycles.

#### 4.3.3 Photothermal activation of 3D-printed LCE structures

Ambulo et al. developed 3D-printable inks for DIW by combining an LCE matrix with liquid metal (eutectic gallium indium alloy, EGaIn) [136]. Due to the electrical paths of the liquid metal, the fabricated ink was electrically conductive. LC mesogens were aligned automatically by the shear force during printing. Although the actuation strain was decreased compared to pure LCE inks, there was an increase in the elongation modulus for inks containing liquid metals. With 71 wt% of liquid metals, the fabricated ink showed the photothermal effect in the NIR region. They printed bilayer bending structures by printing a bottom layer at the 0° direction and an upper layer at the 90° direction. Under NIR illumination, the printed bilayer structure was reversibly bent and recovered. With even higher wt% of liquid metals, the printed structure could also be controlled by the electric voltage bias; due to the electrical paths of the liquid metal, the fabricated ink showed electrical conductivity with a resistance range from 0.0038 to 0.035 Ω. The printed structure was deformed by Joule heating at 1.6 V.

### 4.4 Light activation of 3D-printed microstructures

#### 4.4.1 Photothermal activation of 3D-printed microstructures

**Figure 11:**
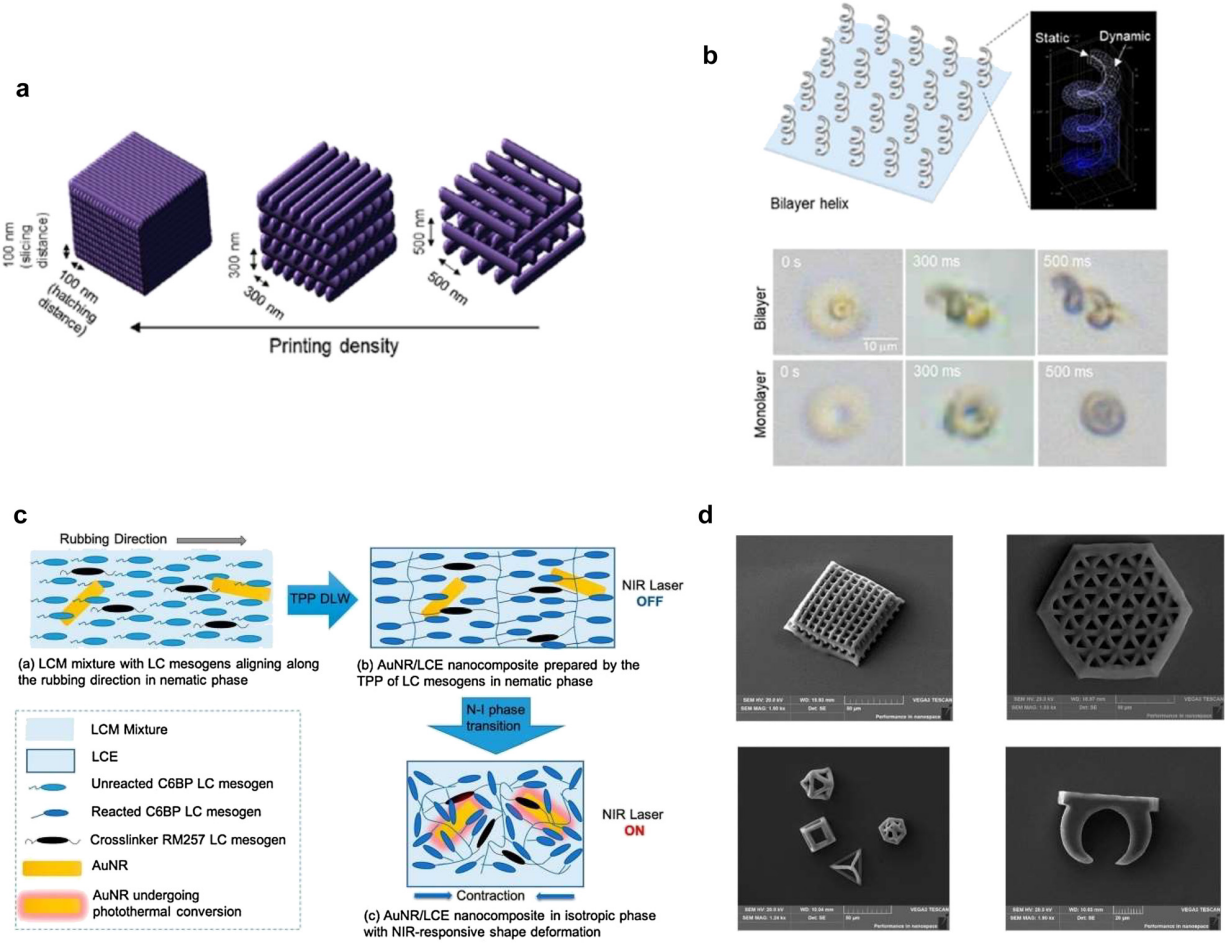
Photothermal activation of 3D-printed microstructures. *DLW of Hydrogel microstructures*: (a) schematic for printing density control via hatching/slicing distance variations from 100 to 500 nm. (b) Schematic of a bilayer helix and phase-contrast images of bilayer and monolayer helices after the laser illumination of 300 and 500 ms. Adapted from Ref. [137]. Copyright (2020) ACS Publications. *DLW of LCE microstructures*: (c) mechanism of light-induced reversible shape morphing in AuNR/LCE nanocomposite structures. (d) SEM images of AuNR/LCE microstructures (woodpile structure, hexagonal photonic crystal structure, wireframe structure, micro-clamp structure). Adapted from Ref. [138]. Copyright (2019) ACS Publications.

DLW based on multi-photon absorption polymerization can allow sub-micrometer resolutions and can be useful for 3D printing of microstructures. Light-activated hydrogel microstructures, for example, were fabricated using DLW [137], where the resolution of DLW was improved down to 100 nm using an appropriate crosslinker. PNIPAm hydrogels were crosslinked with different resolutions. Controlling slice and hatch distances (Figure 11a), the swelling and deswelling factors of the printed structure were studied. Intriguingly, the printed structure with 100 nm distance showed no deswelling at increased temperature. On the other hands, the printed structures with lower resolution showed deswelling features at increased temperatures. Exploiting this feature, a bilayer helix structure was fabricated (Figure 11b), where the bottom layer was printed with 100 nm resolution and the upper layer with 500 nm resolution. Printed bilayer hydrogel structures were actuated in water at different temperatures. To make the hydrogel structure photo-responsive, AuNRs were dispersed in water. NIR-absorbable AuNRs induced a temperature increase in the bilayer helix structure within 500 ms. Figure 11b shows the schematic of bilayer helices and compares the actuation of bilayer and monolayer layer helices. Under NIR laser illumination, the bilayer helix was collapsed within 500 ms and reversibly actuated. In contrast, the monolayer helix was actuated in an isotropic manner only.

Chen et al. fabricated photothermally actuating LCE microstructure using DLW (Figure 11c and d) [138]. Figure 11c shows the fabrication steps for the LCE microstructure. Unlike the study above, they incorporated AuNRs into un-crosslinked LC monomer solution before conducting two photon polymerization (TPP). Because there is no shear force during the printing in the case of TPP DLW, a rubbed polyimide layer was prepared to align LC mesogens and AuNRs. After alignment, TPP was conducted to create microscale structures. Printed structures can shrink due to the isotropic conversion of the LC mesogens under NIR laser illumination. After the laser is off, it can recover its original state due to the re-alignment of the LC mesogens. They also printed various microstructures including woodpile, hexagonal photonic crystal, wireframe, and micro-clamp structures. Figure 11d shows the SEM images of such microstructures created by TPP DLW.

#### 4.4.2 3D printing of light-driven micro-robots

**Figure 12:**
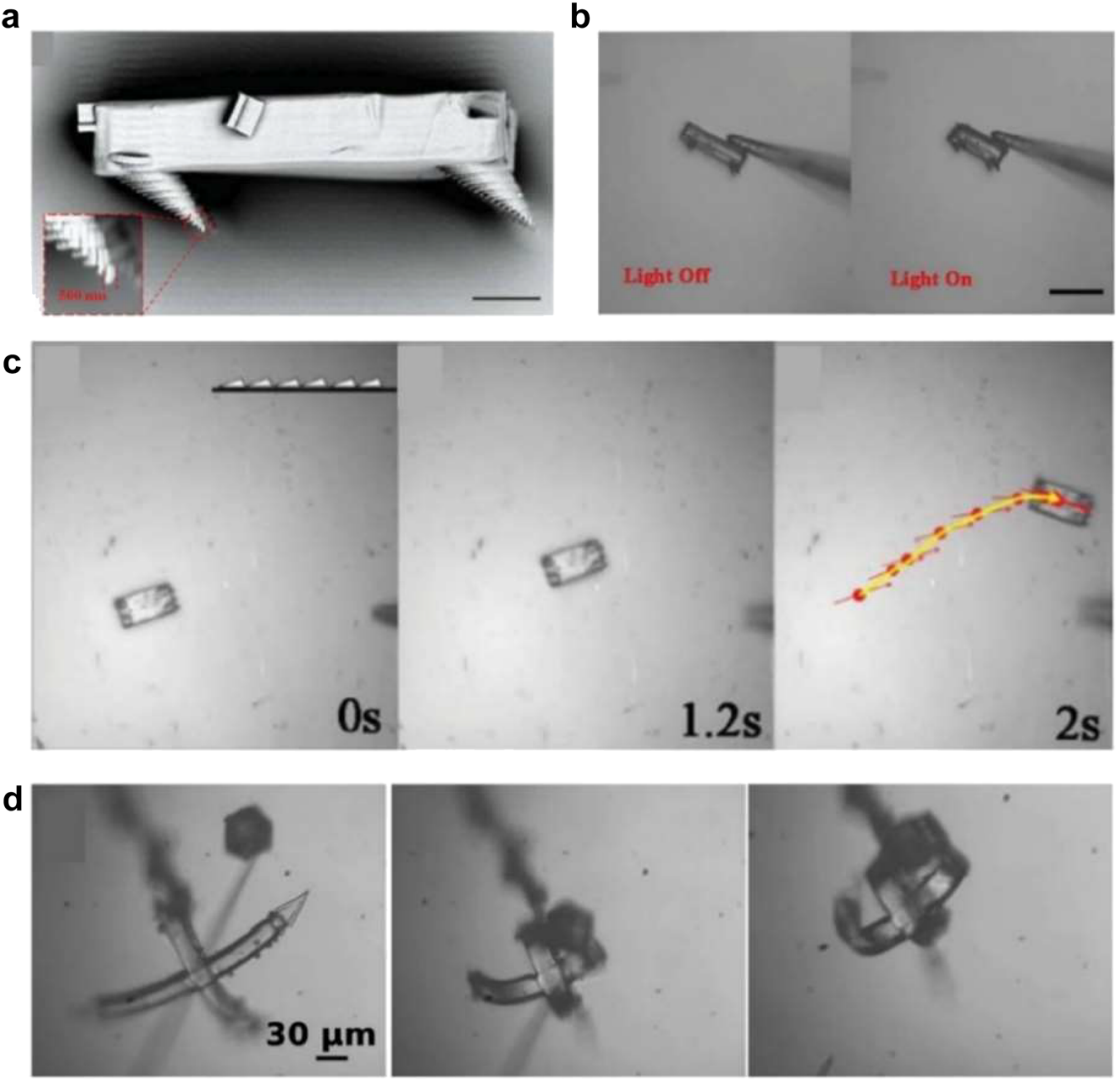
DLW of light-driven micro-robots. *Micro-walker*: (a) SEM image of an LCE micro-walker. Tilted four legs induce walking motion (scale bar: 10 μm). (b) Actuation of the micro-walker under 532 nm laser illumination (scale bar: 50 μm). (c) Walking motion of the micro-walker on a nanograting surface. Inset indicates surface morphology. Adapted from Ref. [139]. Copyright (2015) John Wiley & Sons. *Micro-gripper*: (d) SEM images of a micro gripper. The gripping motion of a micro-cubic structure is demonstrated under 532 nm laser illumination. Adapted from Ref. [140]. Copyright (2017) John Wiley & Sons.

3D printing was also used to fabricate light-driven micro-robots. Zeng et al. fabricated light-activated microscale walkers using DLW [139]. They introduced dyes in an LC monomer mixture. In this work, the mixture was transparent at the DLW photopolymerization wavelength. Because the absorption peak of the dye is around 530 nm, a chopped 532 nm laser could increase the temperature of the microscopic walker to around 100 °C. Figure 12a shows the SEM image of the fabricated LCE micro-walker. It is composed of a 60 × 30 × 10 μm^3^ LCE body and four rigid tilted legs. Figure 12b shows the actuation images of the fabricated microscopic walker under 532 nm laser illumination. Due to photothermal heating, its length is contracted by up to 20%. Reversible contraction and recovery through chopped laser illumination generate elastic energy, which is transformed into the kinetic energy for micro-walking. Figure 12c shows the SEM images of the microscopic walker on a nanograting substrate at a velocity of 380 μm/s.

Martella et al. fabricated a light-activated microscale gripper using DLW [140]. They used dye-doped LCEs to actuate the gripper under 532 nm laser illumination using the photothermal method. Splay-aligned LC mesogens were turned into the isotropic state under laser illumination. Figure 12d shows the SEM images of the micro-griper showing a gripping motion. It takes 30 ms to complete the closure of the micro-gripper, and it takes 25 ms for the gripper to be relaxed.

### 4.5 Multicolor 4D printing for selective heating and photothermal activation

**Figure 13:**
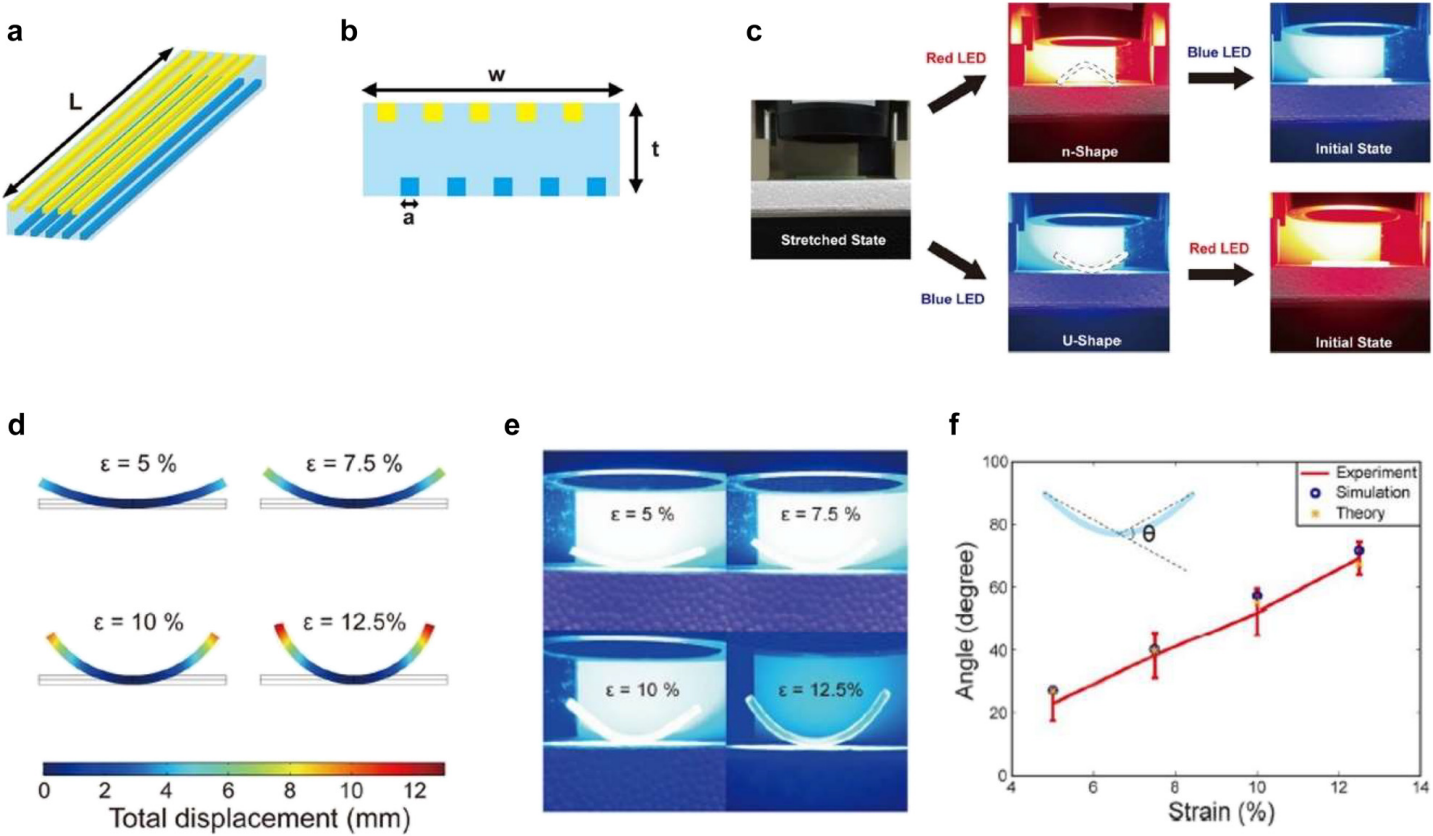
Multicolor 4D printing for remote light actuation. (a) Schematic of multicolor bending structures. (b) Side view of printed structure. (c) Different shape morphing behavior of the structure with different LED color sequences. (d) FE simulation results under different strain values. (e) Experimental results under blue LED illumination. (f) Comparison of the bending angle between experiment, simulation, and Timoshenko beam theory. Adapted from Ref. [141]. Copyright (2020) Springer Nature.

Color-dependent selective heating can be used for sequential shape morphing. Multicolor 4D printing for selective heating and remote activation was demonstrated using multi-material PolyJet 3D printing (Figure 13) [141]. Blue and yellow SMPs were printed as fibers in a rubbery matrix (Figure 13a and b). This multicolor composite material allows color-dependent remote actuation via selective heating of SMP fibers. Figure 13c shows different morphing behavior of the printed composite structure. A thermomechanically stretched structure can be deformed into different shapes depending on the incident light color. Under the red LED illumination, only blue SMP fibers strongly absorb incident light and photothermally recover their length, while yellow SMP fibers do not absorb red light strongly. The length difference between the upper and lower SMP fiber layers makes the structure bent downward (‘n-Shape’). The following blue LED illumination makes the structure flattened again via photothermal heating and shape recovery of yellow SMP fibers too. On the other hand, illuminating blue LED before red LED makes the structure bent to u-Shape first and then flattened (lower panel in Figure 13c). Multicolor SMP composites were also employed to demonstrate multistep hinge structure and sequential folding following the color sequence of light illumination.

To analytically model the photothermal actuation of multicolor SMP composites, the shape recovery ratios of blue and yellow SMPs was first measured at different temperatures. In addition, the temperature of SMP fibers under blue and red LED illumination was measured too. The measured data were used to calculate bending angles following Timoshenko beam theory [63]. Finite element (FE) simulations of bilayers were also conducted to calculate bending angles and compared to analytic calculations. Figure 13d shows FE simulation results for several programming strain values. As the strain ε in the thermomechanical programming step increases, the maximum bending angle of the beam increases too. Figure 13e shows the pictures of the corresponding experiments under blue LED illumination. The bending angles from experiments, analytic calculations, and FE simulations are compared, and they are found to agree with each other well (Figure 13f).

## 5 Conclusions and outlook

As light has played a key role in conventional planar photolithography, light is also essential in many 3D printing processes such as photopolymerization or laser sintering. In addition, nonlinear multi-photon absorption enables that the printing resolution of DLW can reach the sub-micrometer scale (down to ∼100 nm) [142]. As discussed in the current review, light can also play a crucial role in 4D printing. 4D printing can realize active or reconfigurable structures with desired structural and functional changes. The shape of 3D-printed structures can be transformed in response to external stimuli. Among various stimuli, light illumination provides distinct advantages for active devices. Light can induce structural changes remotely and selectively. For example, the spatial location, wavelength (or color), and polarization of light can be selectively chosen to realize highly precise and sequential actuation in 3D-printed complicated structures. In addition, it is also possible to locally heal damaged parts of 3D-printed structures by light illumination.

In this article, we have presented a comprehensive review of the light activation of various structures. We focused on the design and recent demonstration of remote light activation based on photochemical and photothermal mechanisms. In particular, we detailed the photothermal activations based on nanomaterial composites. We have looked at how such light-activation mechanisms were used for 3D-printed, active structures from the millimeter scale to the sub-micrometer scale. Table 2 summarizes representative examples for the light activation of 3D-printed structures.

**Table 2:** Light activation of 3D-printed structures.

Author	Mechanism	Printing method	Demonstration
Ceamanos et al. [90]	Photochemical	DIW	Reversible bending
Hagaman et al. [91]	Photochemical	DIW	Reversible bending with fast actuation (∼5s)
Davidson et al. [92]	Photochemical	DIW	Reversible shape morphing structures
			Lock-in behavior under UV light illumination
Yang. et al. [21]	Photothermal	FDM	Shape morphing under sunlight
Zhang. et al. [130]	Photothermal	DIW	Self-healing due to dynamic covalent bonding
			Shape memory behavior under NIR illumination
Bi et al. [131]	Photothermal	FDM	Enhanced thermal conductivity, mechanical properties
			Shape memory behavior under NIR illumination
Cui et al. [132]	Photothermal	DIW + FDM	Shape memory behavior of a brain structure under NIR illumination, improved cell differentiation due to optoelectronic characteristics of graphene
Keneth et al. [133]	Photothermal	DLP	Sequential shape memory behavior
Han. et al. [134]	Photothermal	SLA	Biomimetic actuation
Zhang et al. [135]	Photothermal	DIW	Multi-stimuli (heat, vapor, light) induced reversible shape morphing behavior
Ambulo et al. [136]	Photothermal	DIW	Multi-stimuli (light, electricity) induced shape morphing behavior
Nishiguchi et al. [137]	Photothermal	DLW	Fast actuating microscale bilayer helix via programming of crosslinking density
Chen et al. [138]	Photothermal	DLW	Reversible microscale shape morphing structure
Zeng et al. [139]	Photothermal	DLW	Microscale walking robot via reversible contraction and relaxation motions under chopped laser irradiation
Martella et al. [140]	Photothermal	DLW	Microscale gripper, reversible grabbing motion actuated by a 532 nm laser
Jeong et al. [141]	Photothermal	PolyJet	Multicolor 4D printing for sequential shape morphing

In addition to shape transformation, 4D printing of light-active materials can also be employed for tunable nanophotonic devices [143]. Nocentini et al. fabricated a photoresponsive waveguide-coupled ring resonator using DLW of azo-compound-doped LCs [144]. To introduce photoresponsivity, an additional cylindrical structure made of azo-compound-doped LCs was added to the ring resonator. Under green laser illumination, the resonance redshifted because of the change in the resonator size. In a modified structure, the refractive-index variation of LCs was induced by light, which led to the blueshift of the resonance. As the illuminating laser power increased, the resonance shift increased. Various tunable nanophotonic and metaphotonic devices can be envisioned. Although the refractive index of polymer materials is rather limited, it can be further increased by incorporating high-index nanoparticles. The gradual variation of material compositions or refractive indices is also possible during multi-material 3D printing.

3D printing technologies are the key to the fourth industrial revolution. Particularly, the rapid development of multi-material 3D printing technologies is introducing many new possibilities and opportunities for 4D printing. New design ideas and materials can be adopted for 4D printing [145], [146], [147]. For example, the concept of multi-stable structures can be extended to the micrometer scale to realize highly controlled actuation or reconfiguration without complicated control systems. Planar microelectronic devices can be reversibly reconfigured to 3D geometries via multistability to enable tunable functionalities [77] or to effectively dissipate heat from devices. Remote and selective control enabled by light illumination can lead to interesting opportunities for micro-robots, biomedicines, implantable bio-medical devices, microelectronic devices, and active nanophotonic devices. In this review, we have introduced and discussed recent ideas and demonstrations in the light activation of 3D-printed structures. We hope that our work stimulates further developments in this emerging field.
